# Superparamagnetic Bead-Based Microfluidic Fluoroimmunoassay Platform for Rapid Ochratoxin A Detection in Flour

**DOI:** 10.1021/acssensors.5c01119

**Published:** 2025-07-30

**Authors:** Daniel López-Puertollano, Charlie Tobias, Jérémy Bell, Antonio Abad-Somovilla, Antonio Abad-Fuentes, Knut Rurack

**Affiliations:** a Chemical and Optical Sensing Division, Bundesanstalt für Materialforschung und -prüfung (BAM), Richard-Willstätter-Str. 11, Berlin 12489, Germany; b Department of Organic Chemistry, University of Valencia, Doctor Moliner 50, Burjassot, Valencia 46100, Spain; c Institute of Agricultural Chemistry and Food Technology (IATA), Spanish Council for Scientific Research (CSIC), Av. Agustí Escardino 7, Paterna, Valencia 46980, Spain

**Keywords:** bead-based assay, fluorescence, immunoassay, microfluidics, mycotoxins

## Abstract

Simplification and reduction of time and costs are the primary goals in the development and use of onsite methods in diagnostics and food safety. To facilitate the transition from laboratory techniques to simple, miniaturized devices, we have developed a modular microfluidic platform. This platform integrates a competitive fluorescence immunoassay on the surface of superparamagnetic beads, serving as a complementary technique to traditional cytometry assays. In the first chip module, a fast competitive reaction (5 min) occurs, after which the particles are retained in the second module. This module consists of a PDMS chip and a permanent magnet, allowing only the fluorescent competitor to reach the detection module. Ochratoxin A (OTA) was chosen as the model analyte for device development, using fluorescein-labeled OTA as a competitor. The system efficiently separates particles, with OTA concentration directly correlated to the amount of fluorescent competitor remaining in solution after the competitive reaction. This innovative setup allows to perform rapid measurements with small sample volumes in a short time (10 min), achieving a limit of detection for OTA of 1.2 μg L^–1^. The system was successfully applied to the accurate determination of OTA in wheat flour spiked at regulatorily relevant concentrations. Using this device, conventional cytometry immunoassays can be seamlessly transformed into user-friendly, miniaturized analytical methods at reduced cost for applications outside of a laboratory directly at the point of need.

## Graphical abstract

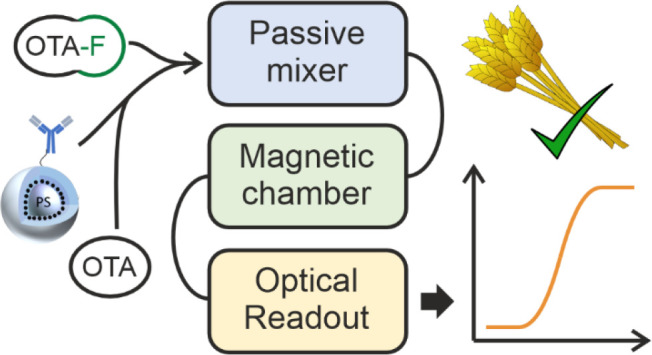

The development of point-of-need systems for (bio)­chemical analysis, which enable rapid onsite measurements with a minimum of manual steps by the user, i.e., ideally only the application or injection of a liquid sample, has gained a lot of interest in recent years due to increasing concerns about food safety or early diagnosis of diseases.^1,2^ In general, microfluidics hold great promise for the development of such systems, with paper- and chip-based approaches being perhaps the most prominent ones.^3–5^ However, whereas paper-based systems have received a tremendous boost in particular by the SARS-CoV-2 pandemic,^6^ chip-based systems have only shown a relatively constant to recessive development during the past 15 years as judged by the published scientific output (Figure S1). On the one hand, this is presumably related to the increased simplicity of paper-based devices, especially in combination with smartphones as readout and data processing devices.^7^ On the other hand, their popularity also relates to the fact that in point-of-care testing (POCT) applications in the health area, quantification is less important than a limit of detection (LoD)-related “Yes/No” result and that such diagnostic tests are mainly developed for a single disease/biomarker, the patient’s symptoms already narrowing down the diseases in question. In other areas such as food and feed, environmental monitoring, or security (e.g., drug detection), quantification or the potential for multiplexing of such onsite analytical systems is much more important.^8,9^ Here, embedded lab-on-a-chip (LoC)-type systems are potentially better suitable because they possess a higher modularity and functionality with respect to tailoring of the assay workflow together with a low reagent consumption.^10–12^

Microfluidic LoC systems unfold their potential perhaps best in combination with sub- or few micrometer-sized particles as carriers for the (bio)­chemical recognition and/or indication elements because in addition to binding, such beads can be endowed with various functions such as codes for analyte identification or magnetic properties for improved handling during separation or washing steps.^13–15^ Especially, their combination with immunoassays, taking advantage of the specificity of antibodies, is a powerful approach that has been successfully applied to the detection of bacteria, viruses, or small-molecule analytes.^16–20^ In the case of small-molecule analyte immunodetection, a reporter/competitor is often needed. Several examples have been described, including electrochemical, colorimetric, or fluorescence detection.^19,21–23^

One of the main applications of analytical point-of-need systems is food safety. In this regard, mycotoxins are a constant concern as evidenced by the recently revised European Union (EU) regulation that sets maximum levels for the presence of these biotoxins in various foods.^24^ One of the mycotoxins included in this regulation is ochratoxin A (OTA). This harmful toxin is mainly produced by *Aspergillus* and *Penicillium* species and can be found in cereals, spices, coffee, dried fruits, or grape-derived products. In 1993, it was classified into group 2B by the International Agency for Research on Cancer (IARC), that is, as possibly carcinogenic to humans. Moreover, nephrotoxic, teratogenic, and immunotoxic effects have also been attributed to OTA.^25^ High-performance liquid chromatography (HPLC) with fluorescence detection, after immunoaffinity column cleanup, is the preferred method by the European Committee for Standardization.^26^ Nevertheless, this method, although highly sensitive, requires expensive and complex equipment, time-consuming procedures, complex sample cleanup, and highly trained personnel. Such a standard method is unsuitable for milling companies, for example, which have to decide on the spot and within tens of minutes whether or not to accept raw materials such as grains or cereals due to mycotoxin contamination, which can have major industrial and economic consequences.^27^ Alternatively, receptor-based assays and in particular immunoassays are user-friendly, rapid, and potentially high-throughput analytical methods that have also been explored for OTA^28–30^ but often have drawbacks in terms of ease of use (complex liquid handling), assay time (long incubation times), regeneration (limiting high throughput), and discontinuous workflows and are therefore not easy to automate when used in microfluidic formats.^31–41^ In an attempt to simplify the general workflow toward mix-and-detect, we recently reported on the development of a bead-based cytometer assay for wheat flour.^42^ However, since cytometers are not ideally suited for point-of-need analysis, we report here on a microfluidic assay for OTA with quick, ready-to-inject readout and simple equipment, transforming our previous work into an onsite format.

The present approach is complementary to a traditional cytometer assay as it uses the same mix-and-detect immunoassay principle yet detects the excess of the free fluorescent tracer instead of the bead-bound one, this approach utilizing an inline competition step between the analyte OTA and a fluorescently labeled OTA tracer for the monoclonal anti-OTA antibodies immobilized on the surface of superparamagnetic core–shell particles. Instead of a reciprocal correlation of measured fluorescence signal and mycotoxin concentrations, this assay yields a direct correlation of both quantities. This approach is enabled by the superparamagnetic properties of the particles that allow for straightforward separation and removal of the bead-bound tracer from the reaction mixture by a magnet. Figure 1 introduces the conceptual strategy. The fluorescence in the supernatant is then measured with a simple and miniaturized fluorescence module employing a laser diode and a miniaturized detection unit with a photomultiplier to afford a signal that is directly proportional to the concentration of the analyte. Using this system, conventional cytometer immunoassays can be easily converted into embedded, cheaper, and easy-to-use devices, for instance, for raw product analyses at flour mills.^43,44^

**Figure 1:**
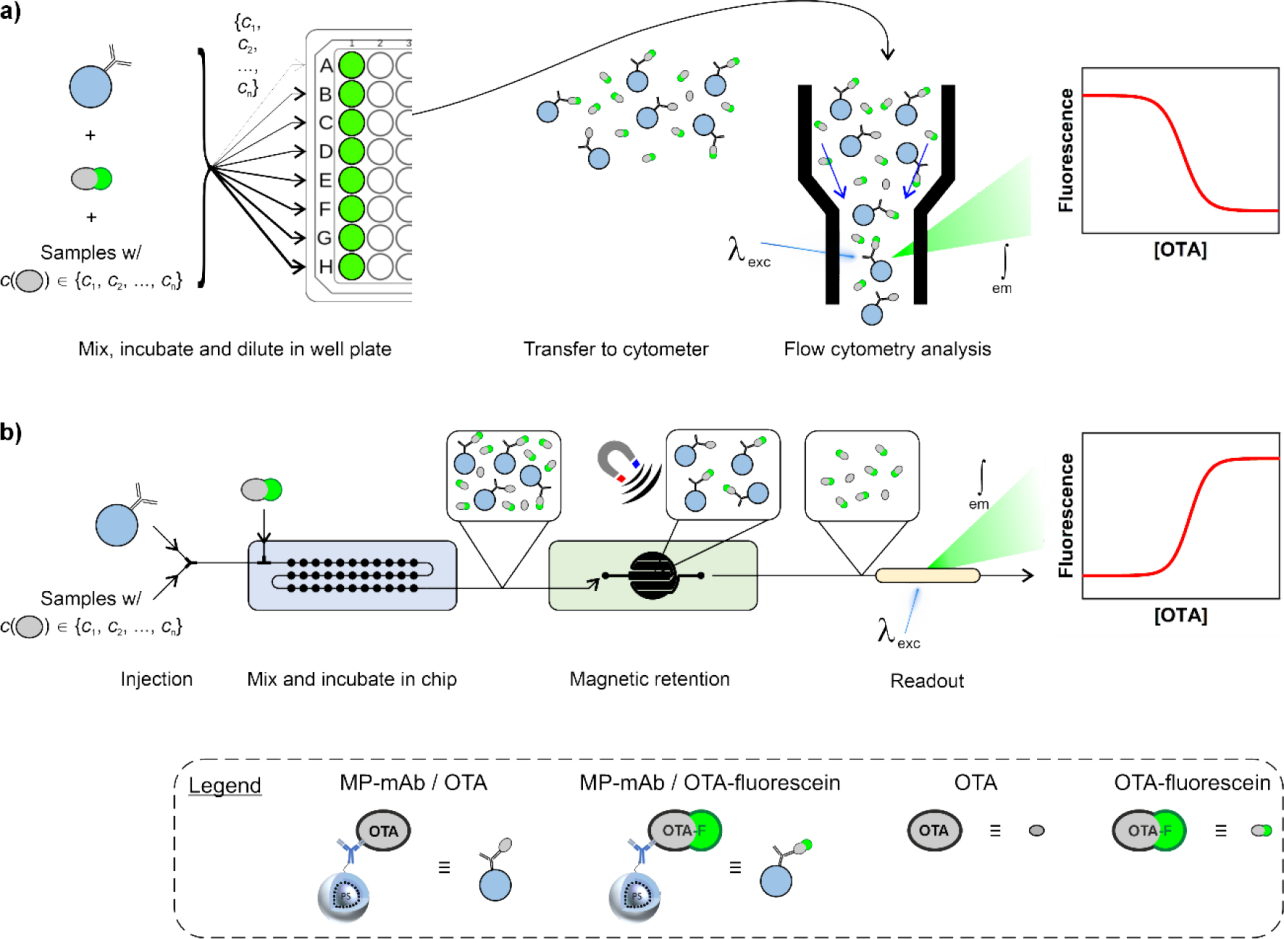
(a) Schematic of the steps for a well plate-based competitive immunoassay with flow cytometer analysis: mixing of solutions containing the mAb-decorated particles, the competitor, and the sample potentially containing OTA, followed by incubation, dilution, and autosampler-assisted transfer into the flow cytometer for scattering and fluorescence analysis. (b) Schematic of the steps for a competitive immunoassay in the modular microfluidic inline platform developed in this work: injection of the particles, the sample potentially containing OTA, and the competitor into the passive mixer module, followed by magnetic particle separation through retention and inline fluorescence analysis of OTA-F. The workflow in (a) corresponds to the data shown in Figure 4 (red), and the workflow in (b) to the data shown in Figure 7a (blue). A complete overview of the workflows used in this work is given in Figure S2.

## Experimental Section

Details on reagents, general techniques, flour sample preparation, and measurement uncertainties are provided in the Supporting Information.

### Microfluidic Platform

Syringe pump stands (DK Infusetek SPC/ZU-I) were employed for injection. Transparent PFA high-purity tubing (inner diameters ID = 0.25 and 0.50 mm) was purchased from PerkinElmer and a pearl chain mixer (Fluidic 658/Topas) from Microfluidic ChipShop. Nonsealed PDMS chips were manufactured in-house (see below). For magnetic separation of the particles, an in-house manufactured sandwich holder was used consisting of two parts, a transparent PMMA plate (top) and an aluminum holder with a small opening below the chip position through which a permanent magnet can be inserted (bottom). To ensure sealing, a standard microscope glass slide and the PDMS chip were pressed between both parts. The magnetic separation was carried out by introducing a cubic neodymium magnet (N35, 8 × 8 × 8 mm, Conrad Electronic) under the aluminum part of the holder. The fluorescence detection setup used a modular cube with a 90° excitation–emission geometry. Excitation was performed with a dot laser diode module (488 nm, 20 mW) from Civil Laser. An excitation bandpass filter (MF475-35, center wavelength CWL = 475 nm, bandwidth BW = 35 nm) and an emission bandpass filter (MF530-43, CWL = 530 nm, BW = 43 nm) were purchased from Thorlabs. The emitted fluorescence was focused through an uncoated plano-convex lens (LA1951, focal length *f* = 25.3 mm) to a photomultiplier module (PMT1001/M), both from Thorlabs. The PMT signal was collected with a USB oscilloscope (Voltcraft, DSO-3074, 200 MHz) and plotted and integrated using OriginLab software (v.9.8.0.200). Calibration curves of the competitive assays were fitted to four- and five-parameter logistic models (eqs S1, S2).

### Microfluidic Chip Fabrication

The top part of the chip consisted of a single channel of 400 μm width and 100 μm height, organized in a circular area of 10 mm diameter (with a channel volume of approximately 8 μL). For the fabrication, 2″ silicon wafer molds were used. The mold consisted of a 100 μm layer of SU-8 2100 resist and was fabricated by a conventional soft-lithography technique using a maskless aligner (Dilase 250, KLOE SAS). For chip manufacturing, a monolayer of hexamethyldisilazane was coated for 20 min on the mold by chemical vapor deposition to ensure soft removal of PDMS when peeling off. A mixture of PDMS and its curing agent at a 10:1 ratio was degassed and poured onto the mold (10 g). Cross-linking of PDMS was performed in an oven for 15 min at 140 °C. Then, the PDMS layer was peeled off, edges were cut using a knife, and the inlet and outlet were obtained by punching 1.5 mm holes. The bottom part of the chip consisted of a standard microscope glass slide.

### Antibody Immobilization

In a 2 mL Eppendorf vial, 10 μL (1% w/v) of a suspension of the superparamagnetic particles (see Section III, Supporting Information) in EtOH was dispersed in 100 μL of MES buffer. Then, 80 and 160 μL of freshly prepared solutions of EDC and sNHS, both at a concentration of 1% (w/v) in MES buffer, were added. After incubation of the mixture for 15 min on a rotator plate (5 rpm), the particles were centrifuged (3 min, 1000*g*), the supernatant removed, and the particles redispersed in 350 μL of fresh MES buffer. Directly afterward, 20 μL of anti-OTA mAb (1 g L^–1^) in PBS buffer was added to the particles. After overnight incubation at 20 °C, the particles were washed twice with fresh PBS by centrifugation and finally dispersed in 200 μL of fresh PBS to afford a stock solution with a concentration of 0.05% (w/v).

### Competitive Offline Assay

In the competitive offline assay, 30 μL (0.05%) of mAb-functionalized particles was mixed with 160 μL of OTA solutions at different concentrations in PNa buffer and 30 μL (0.05 μM) of the OTA-fluorescein (OTA-F) competitor as a tracer in Milli-Q water. The mixtures were incubated at room temperature for 10 min and then processed. For the confirmation measurements using the cytometer, 10 μL of the mixture was diluted with 90 μL of fresh PNa buffer. For the microfluidic platform, a total of 20 μL of the competition mixture was injected at a flow rate of 20 μL min^–1^, followed directly by the injection of PNa buffer at the same rate to wash the tubing, with a total time of 1 min for signal acquisition.

### Competitive Inline Assay

For the inline assay, the three modules, i.e., a chip mixer for competitive reaction, a PDMS chip, and a detection module, were connected with transparent PFA tubing (ID = 0.25 mm). PFA tubing (ID = 0.50 mm) was only used in the detection module to increase the liquid cross section at the detection position and gain sensitivity. mAb-functionalized particles (0.05%) and increasing concentrations of OTA samples in PNa buffer were mixed through a Y-cross junction at 19.2 and 3.6 μL min^–1^, respectively. The outlet of the Y-cross junction was connected to one of the inlets of the pearl chain mixer, and the other inlet was used to inject OTA-F (0.05 μM) in Milli-Q water at 1.2 μL min^–1^. The total incubation time in the mixer module under these flow conditions was 5 min. The resulting mixture was injected into the magnetic separation module through another Y-cross junction for which the second inlet of the junction was used for washing of the PDMS chip with PNa buffer at 24 μL min^–1^. Upon particle retention in the PDMS chip, the signal from the flowing supernatant was acquired for 3 min using the detection module.

## Results and Discussion

Bead-based microfluidic assays are particularly attractive when the reversible magnetization of superparamagnetic particles is utilized. These particles remain nonmagnetic and well-dispersed in solution but become magnetized when exposed to an external magnetic field. These properties enable reversible control of bead movement, facilitating efficient separation, washing, and detection steps in miniaturized analytical systems. In this work, we have developed a simple microfluidic system that integrates superparamagnetic beads for the detection of small-molecule analytes such as OTA and represents a cost-effective alternative to conventional benchtop cytometer assays for use outside of a dedicated laboratory environment. The fluidic setup was designed to quantify the fluorescent OTA competitor (OTA-F) in solution, with the magnetic beads enabling the separation of both a bound analyte and bound OTA-F. Although the system has been successfully used for the analysis of real flour samples, this study focuses on the microfluidic quantification of OTA in aqueous extracts. Automated sample preparation was not integrated, as rapid extraction methods for OTA are well-established^45^ and often require adaptation to specific sample types.

### Particle Design and Antibody Selection

Hybrid polystyrene-core silica-shell beads have been employed for the detection of several analytes in cytometric and microplate reading systems because of their versatility, high-throughput potential, and the simplicity of the respective assays, relying on immunoanalytical as well as DNA binders.^42,46–53^ In this study, the hybrid superparamagnetic variants of these materials previously described by us have been used,^42,54^ consisting of a polystyrene core, a first thin silica shell that contains individual and small patches of superparamagnetic iron oxide nanoparticles, and a second silica shell, which insulates the inner parts against the environment and can itself be functionalized.

The magnetic properties allow the separation of the particles from the other reagents, opening the possibility to work separately with the signal of both particles and the supernatant. Moreover, the outer silica surface facilitates functionalization through silane chemistry, allowing the attachment of antibodies or other receptor structures. Here, the surface was first functionalized with amino groups using (3-aminopropyl)­triethoxysilane (APTES) and later converted into carboxylic acid groups using succinic anhydride. Carboxylic acid groups were then activated via EDC/sNHS chemistry in aqueous solution for the attachment of monoclonal anti-OTA antibodies, ochratoxin A or OTA being the model analyte of this work. The particles used here were partly from previous batches and partly newly prepared and showed identical features within the low uncertainty as described in detail in ref 42. Figure 2a shows a representative SEM image, see refs 42 and 54 for comparison, and representative results of the cytometric quality checks are given in Figures S3 and S4. Regarding the antibody selection, the monoclonal anti-OTA antibody e#115 was chosen for the present work. This antibody was previously generated by our group,^30,55^ using the native carboxylic acid group of OTA for the protein–hapten conjugate used for its production (hapten e) and making it suitable for use with the commercially available OTA-fluorescein (OTA-F) competitor (Figure 2b). It showed no cross-reactivity with unrelated mycotoxins that can be commonly found in flour, thus selectively recognizing the ochratoxin family (Figure S5). Additionally, in our previous work, we observed that this combination of the monoclonal antibody and attachment method (Figure 2c) afforded the highest fluorescence signal in the noncompetitive cytometer assays,^42^ promising also the most pronounced fluorescence changes for the tracer assay attempted here.

**Figure 2:**
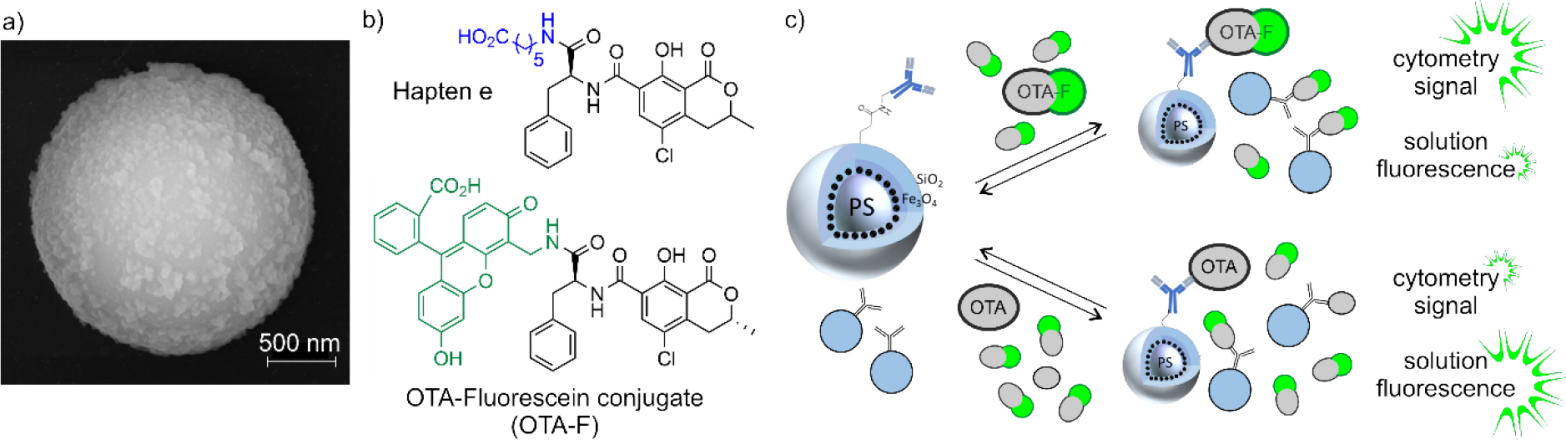
(a) SEM image of a superparamagnetic hybrid polystyrene-core silica-shell bead. Chemical structures of (b) hapten e used for antibody generation (top) and OTA-fluorescein (OTA-F) conjugate as a competitor (bottom). (c) Schematic of the competition mode. In the absence of OTA, only OTA-F binds to mAb-functionalized particles (top) whereas in the presence of OTA, a proportional amount of OTA-F remains unbound in solution. While in the upper case, a high signal is recorded in the cytometer, in the lower case, a strong signal is recorded for the solution, which is symbolized by the different sized halos.

### Fluorescence Detection Module

The detection module was constructed based on the fluorophore used as a label for the competitor, i.e., fluorescein. The absorption and emission spectra of OTA-F were recorded in PNa buffer, presenting a maximum of absorption at 491 nm and a maximum of emission at 510 nm (Figure 3a). Therefore, a 20 mW laser diode emitting at 488 nm was selected as the light source for excitation. A bandpass filter (CWL = 475 nm, BW = 35 nm) was placed in the excitation beam between the laser and the sample to avoid higher wavelengths to reach the sample. Samples were injected via syringe pumps through transparent PFA high-purity tubing (ID = 0.25 mm) and using a low-pressure Y-cross junction. An additional inlet was included to wash the tubing with PBS between sample injections. A check valve was included between the sample syringe and the Y-cross to avoid back flows and sample dilution during tubing washing. PFA high-purity tubing (ID = 0.50 mm) was used in the outlet of the Y-cross junction to increase the optical path of the sample during transit in the detection module. Sample excitation was carried out by spot excitation in an in-house printed sleeve. Finally, the emitted signal was collected and focused through an uncoated plano-convex lens (*f* = 25.3 mm) and a bandpass filter (CWL = 530 nm, BW = 43 nm). Due to the low concentration of the competitor used for the assay, a sensitive photomultiplier module integrating current to voltage conversion was used as the detector. The analog voltage signal from the PMT was acquired and transformed into a digital signal with a USB oscilloscope (Figure 3b). The analyte-related fluorescence intensity signal *I*_F_ was integrated during the transit time of the competitor in front of the detector, and the area was plotted as a function of OTA concentration.

**Figure 3:**
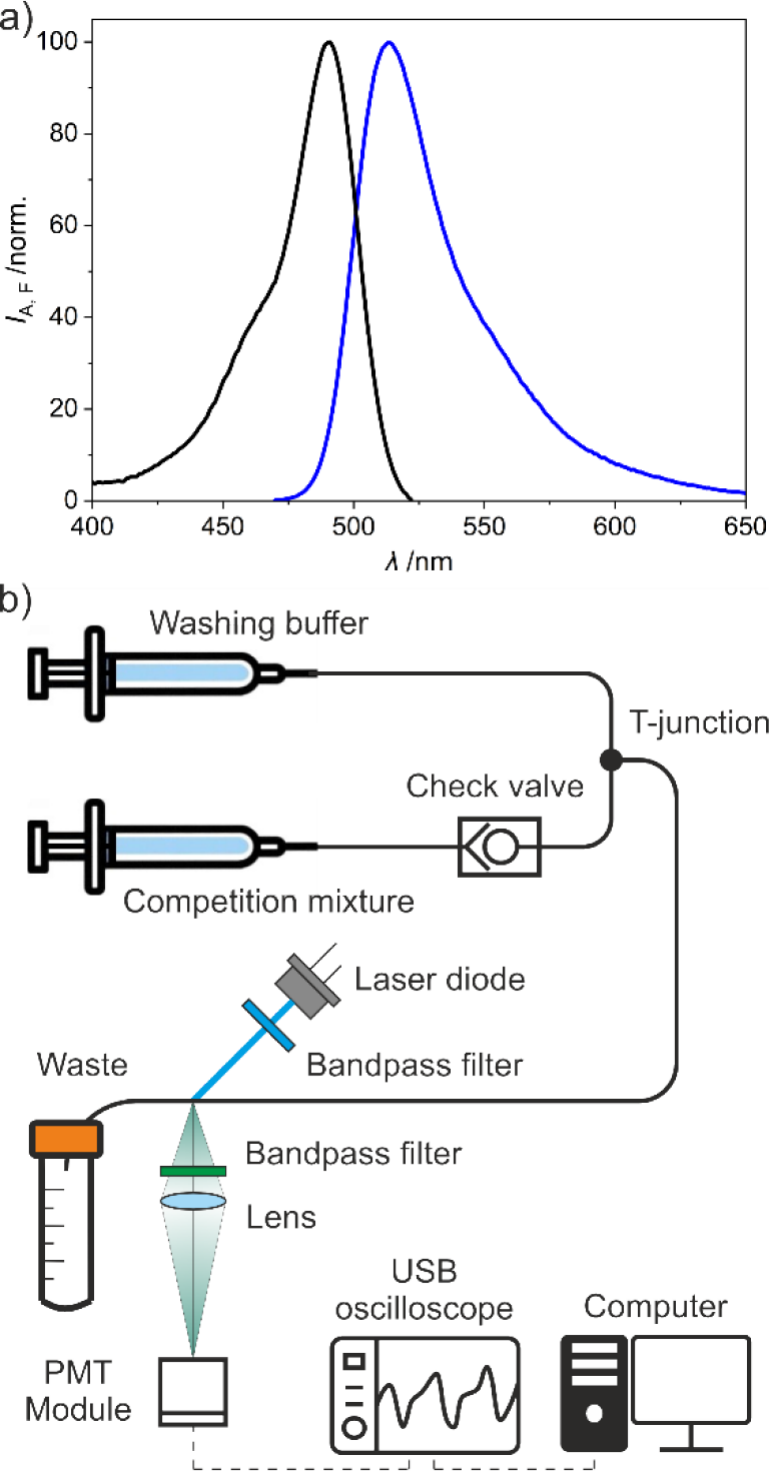
(a) OTA-F absorption (black) and emission (blue) spectra; (b) fluidic module configuration: syringe pumps for injections, with a check valve on the competition mixture channel, and readout of the fluorescence signal from the solution in the tubing with analogue/digital conversion.

To test the applicability of this inline readout setup, a competitive assay was performed in a well plate by mixing several OTA standard solutions with the particles and a constant concentration of OTA-F. After 10 min of incubation, a sample of each mixture was taken out and diluted with PNa buffer for validation of the assay by cytometry and the rest of the suspension was centrifuged to remove the particles and then injected into the fluidic system (for workflows, see Figure 1a and Figure S2a,b). As shown in Figure 4, a decrease is observed in the fluorescence signal measured by the cytometer when the concentration of OTA increases since less OTA-F competitor is bound to the antibodies on the particles’ surface. Accordingly, when the supernatant was injected into the fluidic setup, the opposite effect was obtained: an increase in fluorescence with increasing analyte concentration, because the more OTA is in the assay mixture, the more OTA-F competitor will remain in solution. Under these conditions, the cytometer assay showed an IC_50_ value and a limit of detection (LoD) of 5.7 and 0.1 μg L^–1^, respectively (Table S1). For readout with the miniaturized detector, the calibration plot showed an IC_50_ and LoD at 24.3 and 1.0 μg L^–1^, respectively. As can be seen in Figure 4, in the absence of OTA and at low OTA concentrations, the voltage signal still shows a sizable reading of ca. 130 mV, i.e., does not reach complete inhibition for the fluidic detection, mainly because OTA-F is used in excess in the competition reaction. Additionally, this competition is an equilibrium with three components, i.e., the antibody, analyte, and competitor, so unbound OTA and OTA-F will always be present in solution.

**Figure 4:**
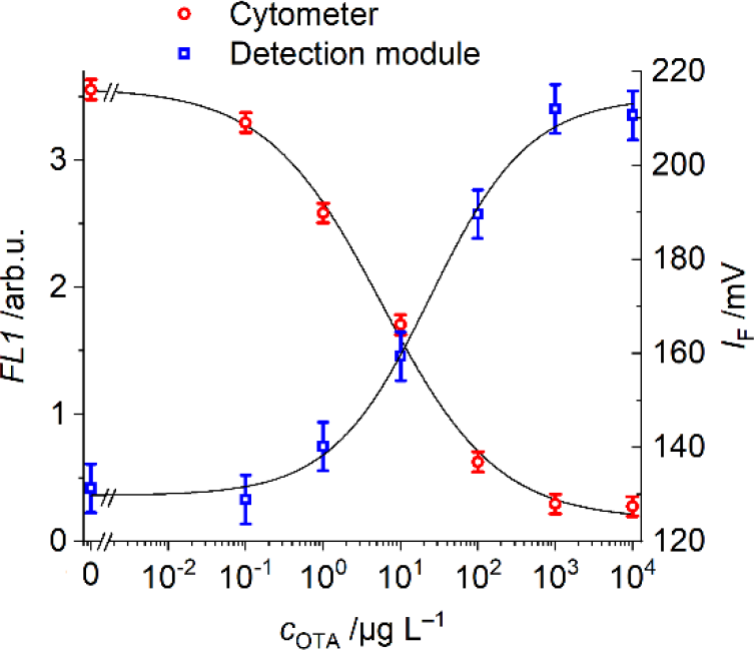
Calibration plots for OTA in water samples after a competitive assay in offline well plate-based sample processing using cytometry analysis (red) and the miniaturized readout platform after removal of the particles by centrifugation (blue). For schematics of the workflows, see Figure S2a,b.

### Magnetic Particle Retention Module

The magnetic retention module consisted of a two-piece in-house manufactured holder and an unsealed PDMS/glass chip. The top part of the holder was made of transparent poly­(methyl methacrylate) (PMMA), and it was designed with two openings to insert the inlet and outlet tubing into the chip. The bottom part was made of aluminum, and it also presented a small lateral opening to position the neodymium magnet below the chip. The chip consisted of a single channel organized in a circular area of 10 mm diameter with a total volume of approximately 8 μL (Figure 5a). Sealing was ensured by pressing both a PDMS half chip containing the channel structure and a microscope coverslip between the two parts of the holder (Figure 5b). This chip design was selected because it afforded a homogeneous covering of the magnetic field facilitating bead accumulation, especially with the serpentine-shaped channel.^56,57^ Not sealing the chip allowed for complete rinsing of the channel after use, ensuring better day-to-day reproducibility.

**Figure 5:**
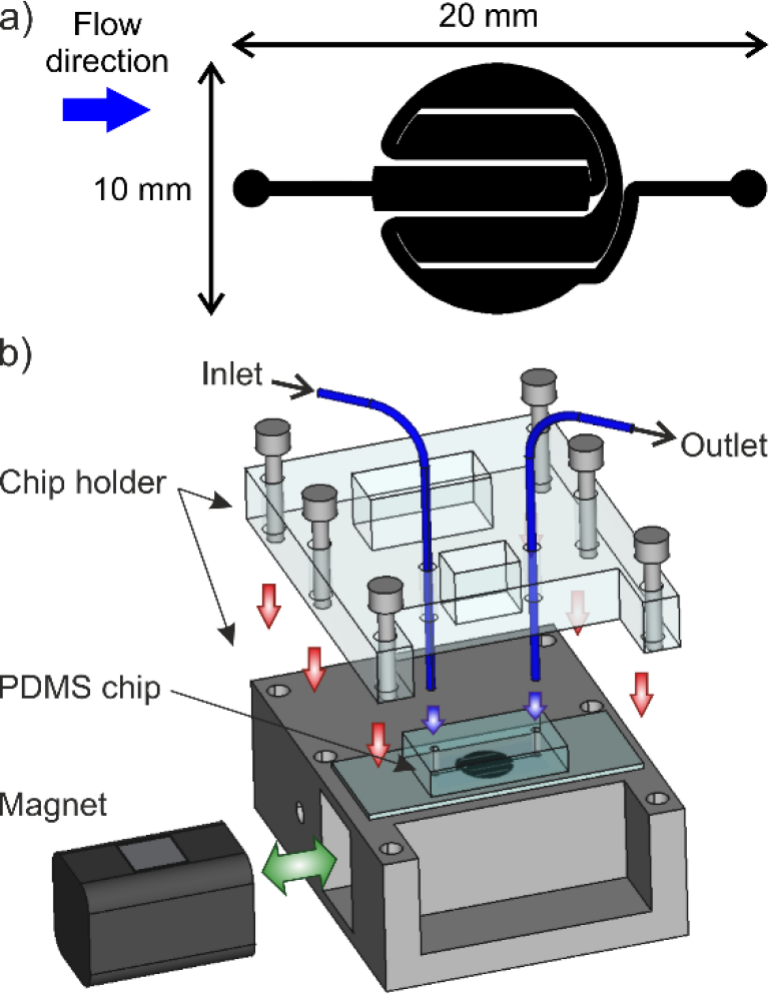
(a) Scheme of PDMS chip channel design for magnetic retention of the particles; (b) assembled module for magnetic separation with a two-part holder, a movable permanent magnet, fluid connection tubing, and a microfluidic chip sealed on a microscope slide by pressure from the holder.

To evaluate the retention capability of the module, a suspension of unmodified particles (0.008% w/v) in PNa buffer was first injected at 20 μL min^–1^ in the presence of a magnetic field. Second, still with the magnet in place, only buffer was pumped through the system and collected to verify particle retention. Cytometer comparison of the collected supernatant containing nonretained particles of the original suspension showed that 85% of the injected particles were successfully retained. Using the same chip for successive retention analysis did not show any significant memory effects since after several 200 μL washings at high flow rates with buffer and without a magnetic field (magnet removed from the chip), all particles were recovered in the first wash as confirmed by cytometry (Figure S7). To further simplify this module, a magnet stack was also built and tested, in which the tubing was passed either through a stack of ring-shaped magnets or between a set of three magnets.^58^ Unfortunately, this approach, as several other magnets that we tried (Table S2), showed less particle retention (20%), probably due to the less strong and homogeneous magnetic field in the tubing, and was not further considered for use with the PDMS chip (Table S2).

In the next step, the combination of the magnetic retention and the fluorescence detection modules was evaluated with a full competitive assay performed as previously described, but without particle separation via centrifugation (see Figure S2c). Each sample containing different OTA concentrations was injected separately, and after completion of each measurement, the magnet was removed from the bottom part of the holder and the chip was washed with PNa buffer between sample injections. Figure 6 shows the obtained calibration plot with an inflection point and LoD at 12.9 and 0.2 μg L^–1^, respectively. These values show that there is no significant signal perturbation by the approximately 15% of nonretained particles present in the supernatant since the values are similar to the ones obtained after particle separation by centrifugation in Figure 4. In addition, Figures S8 and S9 and Section IX, Supporting Information further show that the simple design of the retention module has its strengths for the present assay workflow, i.e., quantifying the unbound competitor in solution, yet that quantitative analysis of particle-bound OTA-F is not reliably possible.

**Figure 6:**
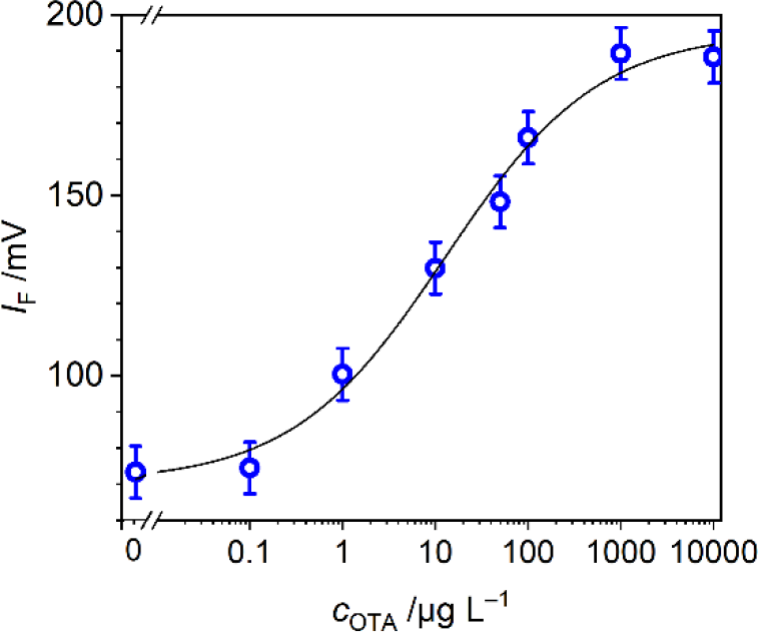
Calibration curve for OTA in water samples after a competitive assay in an offline well plate combined with the magnetic retention and detection modules. For a schematic of the workflow, see Figure S2c.

### Competitive Inline Assay

To evaluate the feasibility of a fully inline competitive assay with the developed device, a passive mixer module was added to the other two modules. First, the mixing efficiency of the selected pearl chain mixer was assessed by injecting particles and samples with different OTA concentrations into one inlet of the mixer using a low-pressure Y-cross junction, while the competitor OTA-F was injected through the second inlet of the mixer. After a transition and mixing time of 5 min, the resulting effluents were collected through the outlet and were measured by cytometry (see Figure S2d). Figure 7a shows that online mixing of the three reagents in the pearl chain mixer chip yielded similar results as the offline mixing in a well plate (Figure 4), demonstrating the efficiency of the mixer chip. It was also observed that a reduction of the flow rates, allowing for longer mixing and incubation times, afforded lower cytometric signals. After cytometric measurement, two populations of particles were observed when lower flows were tested to increase the incubation time to 10 min (Figure S10). Indeed, in accordance with the pearl chain mixing mechanism, stronger chaotic advection and better mixing are obtained with increased flow rates. At low flow rates, the laminar behavior of solutions in microfluidic channels prevents efficient, diffusion-driven mixing. However, increasing the flow rates reduces the efficiency of the magnetic capture in the next module, which is why a residence time of 5 min was chosen in the mixing unit.

**Figure 7:**
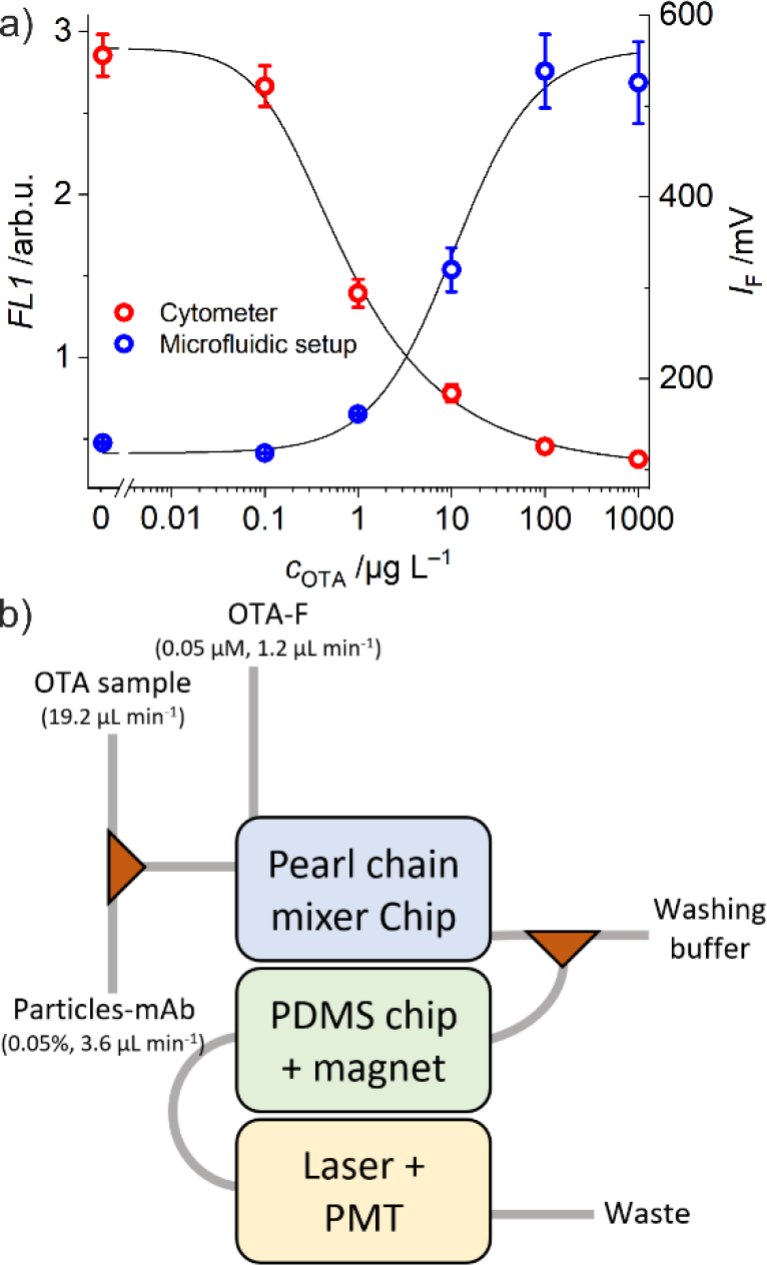
(a) Calibration curves of competitive assays performed with cytometric offline detection (red) and with opto-fluidic inline detection (blue) after mixing and incubation in the pearl chain mixer unit (see Figure 1b and Figure S2e); (b) schematic representation of the final modular opto-fluidic inline platform. A photograph of the laboratory prototype setup is given in Figure S11; representative raw data are shown in Figure S12.

The combination of the three modules (Figure 7b and Figures S11 and S12), i.e., a passive mixer, a magnetic retention, and a detection unit, was finally tested in an assay as described in the Experimental Section (see Figure 1b and Figure S2e). The obtained calibration plot showed an IC_50_ and LoD of 10.3 and 1.2 μg L^–1^, respectively. These values are slightly lower than or equal to those previously obtained with offline mixing (Figure 4 and Table S1), proving the applicability of the modular platform for OTA detection.

If one considers the assay performance of the different setup and device combinations employed in this work via their IC_50_ values, see Table S1 and Section IX, Supporting Information, it becomes obvious that the cytometer was more sensitive than the detection chip due to its superior excitation and detection capabilities (e.g., Table S1, entries #1 vs #2 and #4 vs #5). The pearl chain mixing/incubation chip outperformed well plate-based incubation especially when combined with cytometric detection (#4 vs #1), but also when combined with magnetic retention and chip-based detection (#5 vs #3). Notably, pearl chain mixing with magnetic retention outperformed well plate mixing with centrifugation (#5 vs #2), highlighting the role of the superparamagnetic particles and the magnetic module in enhancing assay sensitivity. Overall, the pearl chain and magnetic retention modules offer strong performance compared to standard lab equipment, while detection sensitivity primarily depends on the optical performance of the instrument, with cytometry offering higher sensitivity at the expense of cost, complexity, and limited portability. Other minor changes between the different combinations arise from specific characteristics of the two types of assays rather than from molecular differences in the biomolecular recognition process. As explained in Section V, Supporting Information, it is known that especially the passive mixing regime of mixers such as the pearl chain module can be responsible for a certain asymmetry often observed in such calibration curves.^59^ However, the calibration curves were well-reproducible for our assays.

The entire assay requires less than 10 min, considering the incubation time of the competitive step (5 min), injection, and signal acquisition (3 min). The LoD reached is in accordance with the limit established by the EC for OTA in food. A two-step extraction, based on the acidic properties of OTA (p*K*_a_ 4.4 and 7.3–7.9),^60^ and subsequent analysis of wheat flour samples spiked with 5 and 20 μg kg^–1^ yielded comparable signals to those obtained in PBS buffer (Figure S13). Although the recoveries were only 66 and 85%, respectively, the performance of the miniaturized assay platform is within EU regulatory requirements.^61^ Moreover, such comparatively low recoveries are also well-documented for commercial immunoanalytical OTA kits and can be attributed to matrix effects of wheat extracts, i.e., they are primarily related to the immunochemistry and not the sensor setup.^62,63^ This problem can be circumvented by using a blank wheat sample,^64^ at best a standardized reference sample, as matrix effects can also vary from batch to batch.^65^ From a sensory point of view, a lower LoD would also help to address this problem. Our laboratories are currently working on solving these issues. This includes generating better monoclonal antibodies and using brighter fluorescent labels, as well as optimizing the respective assay conditions while maintaining low amounts of reagents (antibodies, OTA-F, and particles), thereby not compromising the portability and cost of the device.

## Conclusions

In this work, an integrated microfluidic platform comprising three modules was developed to perform simple and ready-to-inject indirect detection of small organic analytes, such as ochratoxin A. The results presented here proved the possibility of simplifying cytometry measurements through the detection of the fluorescent competitor still in solution after a competitive assay. Building up a modular platform consisting of a pearl chain mixer chip, a magnetic separation, and a fluorescence detection unit, superparamagnetic particles were retained inline, after competitive binding during incubation, using an unsealed PDMS chip in an in-house manufactured holder with a removable magnet. The fluorescent competitor was later detected with a dedicated optical setup based on excitation with a laser diode and signal acquisition with a photomultiplier module. The calibration curves obtained for OTA showed a good correlation between the amount of the competitor and the concentration of the analyte in the solution, enabling the detection and quantification of OTA with an LoD of 1.2 μg L^–1^ and a considerably broad dynamic range of >4 orders of magnitude in only 10 min assay time. Systems with such features are well-suited for raw product analysis in a mill, where the incoming material is heterogeneous and can vary considerably in its degree of contamination (fungal nests). The performance of the automated assay in the miniaturized device can compete well with standard ELISAs and is much simpler and faster than other sensory approaches that have been reported recently (Tables S3 and S4).^28–41^ In particular, the use of beads in a fluidic system is advantageous because multiple samples can be processed sequentially without memory effects, avoiding disadvantages of surface-functionalized chips or single-use assays. The system was successfully employed for the detection of OTA in wheat flour spiked with 5 and 20 μg kg^–1^ of the mycotoxin, which is relevant in the context of current regulations. Furthermore, because the device is a lab prototype, we envision several ways of improving its performance, which, however, should be attempted only with the final application in mind, i.e., which foodstuff or other samples are targeted by the end user. The results presented here contribute to progress in the field of bead-based immunoassays, showing how simplification and miniaturization can lead to lab-on-a-chip devices becoming a valuable tool for onsite measurements, complementing lab-based cytometric approaches while allowing to use the same beads or kits, thus increasing reliability and reproducibility of field and lab methods. Future work will also address the complete automation of the workflow by developing modules for sample preparation, which, however, depend crucially on the final application, the corresponding samples, and the end user’s equipment.

## Supplementary Material



## Notes

Research output in microfluidic methodologies, reagents and general techniques, sample extraction, cytometric controls, laboratory prototype setup, method comparison, and uncertainty budget (PDF)
