# Supplementary material for: Superparamagnetic Bead-Based Microfluidic Fluoroimmunoassay Platform for Rapid Ochratoxin A Detection in Flour
Source: ACS Sens. 2025 Jul 30;10(8):5834–43. doi: 10.1021/acssensors.5c01119 (PMC12379180; doi:10.1021/acssensors.5c01119)
Supplement: Supplementary file 1 [file se5c01119_si_001.pdf]

Supporting Information

Superparamagnetic Bead-Based Microfluidic Fluoroimmunoassay Platform for Rapid Ochratoxin A Detection in Flour

Daniel López-Puertollano, Charlie Tobias, Jérémy Bell, Antonio Abad-Somovilla, Antonio Abad-Fuentes, Knut Rurack*

Chemical and Optical Sensing Division, Bundesanstalt für Materialforschung und -prüfung (BAM), Richard-Willstätter-Str. 11, 12489 Berlin, Germany; Department of Organic Chemistry, University of Valencia, Doctor Moliner 50, Burjassot 46100, Valencia, Spain; Institute of Agricultural Chemistry and Food Technology (IATA), Spanish Council for Scientific Research (CSIC), Av. Agustí Escardino 7, Paterna 46980, Valencia, Spain

knut.rurack@bam.de

Table of Contents

I. Research output in paper- and chip-based methodologies S3

II. Schematics of the various assay workflows used in this work S4

III. Reagents and general techniques S6

IV. Antibody cross-reactivity S10

V. Calibration curves S12

VI. Cytometric control measurements S15

VII. Performance of different magnets in retention module S16

IX. Indirect quantification of OTA through quantification of OTA-F bound by particles S17

X. Incubation time S20

XI. Laboratory prototype setup S22

XII. Wheat flour sample extraction and measurement S24

XIII. Method comparison S26

XIV. Measurement uncertainties S30

XV. References S32

I. Research output in paper- and chip-based methodologies

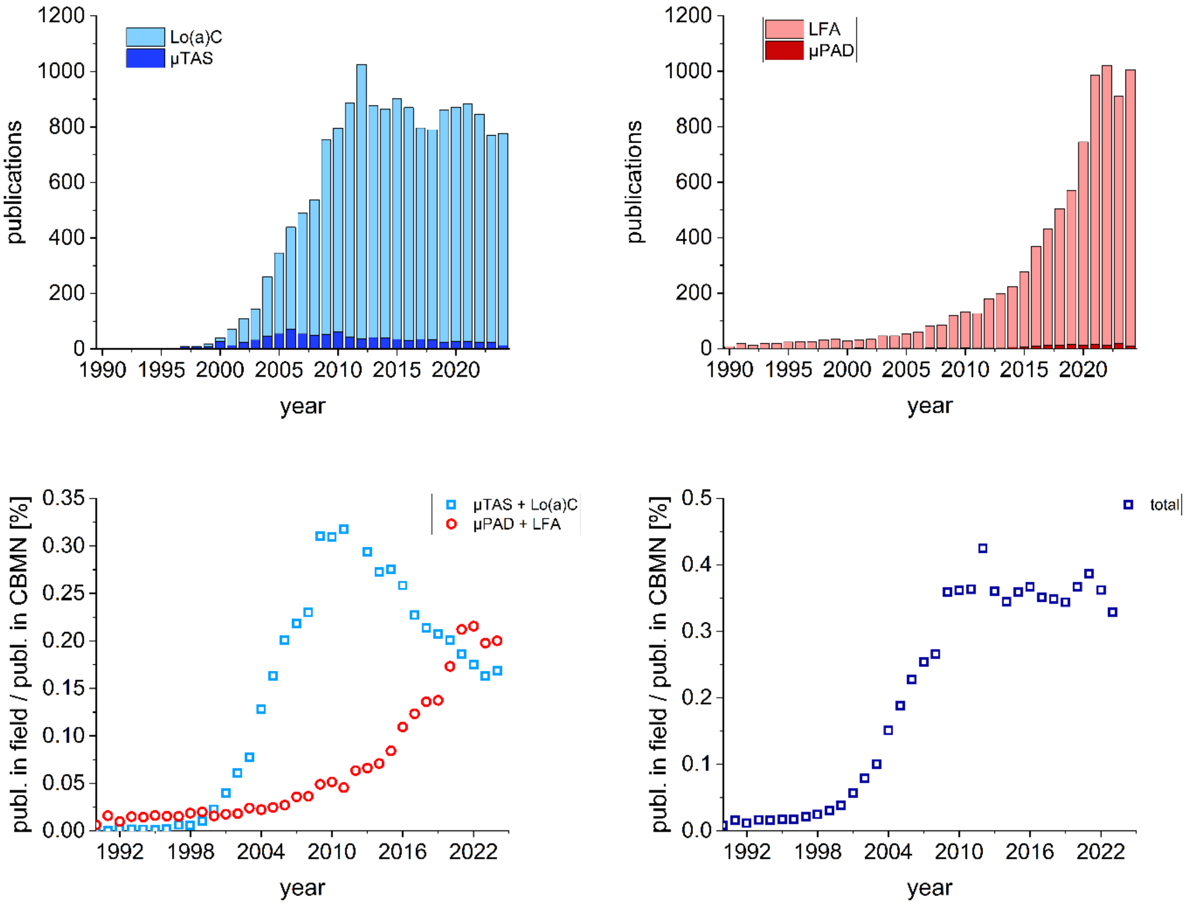

Figure S1. Top: Research articles published in the fields of micro total analysis systems and lab-on-a-chip (left) as well as micro paper analytical devices and lateral flow assays since 1990 according to Web of Science.[1] Bottom: Data of top panels normalized to the general output in chemistry, biochemistry, materials and nanoscience, for both fields individually (left) and for their sum (right), in percent.[2]

---

[1] Search strings: µPAD = "micro paper ("analytical device" or "analytical devices")"; LFA = "lateral flow assay or "lateral flow assays" or "lateral flow immunoassay" or "lateral flow immunoassays" or (flow and immunoassay* and paper* and strip*) or ("immunochromatography" and (paper* or membrane*)) or ((dipstick* or (test strip*)) and immunoassay*) not injection"; µTAS = "(micro or miniaturized) and total and ("analysis system" or "analysis systems")"; Lo(a)C = ""lab-on-chip" or "lab-on-chips" or "lab-on-a-chip" or "lab-on-a-chips" "; only document type "Article" considered.

[2] Web of Science categories considered: "chemistry", "biochemistry & molecular biology", "biochemical research methods", "materials science" and "nanoscience & nanotechnology".

II. Schematics of the various assay workflows used in this work

In addition to the conventional well plate-based cytometer workflow and the final microfluidic workflow developed here, which are shown in Figure 1 of the main text, Figure S2 collects additional assay workflows that were used during the development of the competitive microfluidic immunoassay. The column on the right lists the figures in which data obtained with the single workflows are reported.

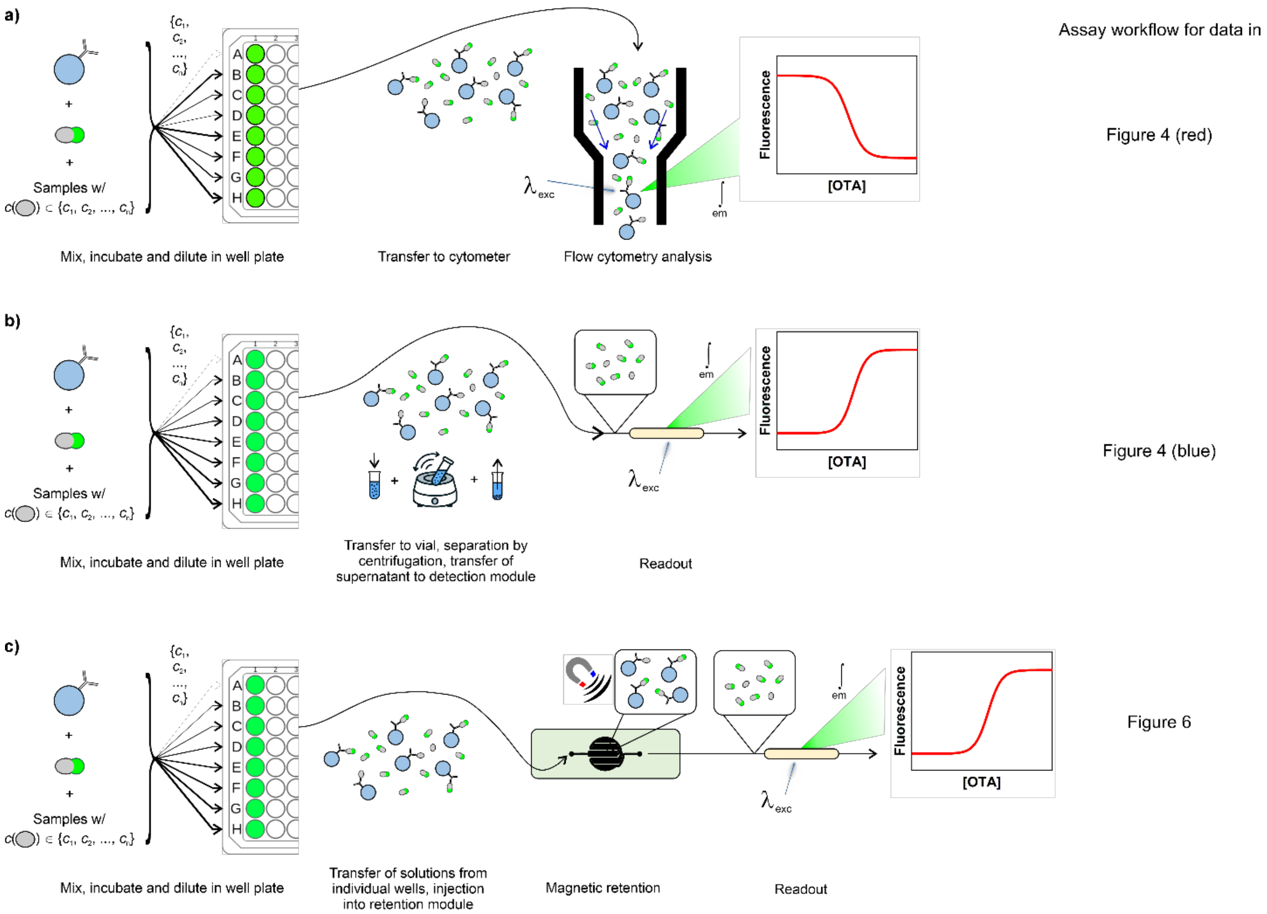

Continued on next page.

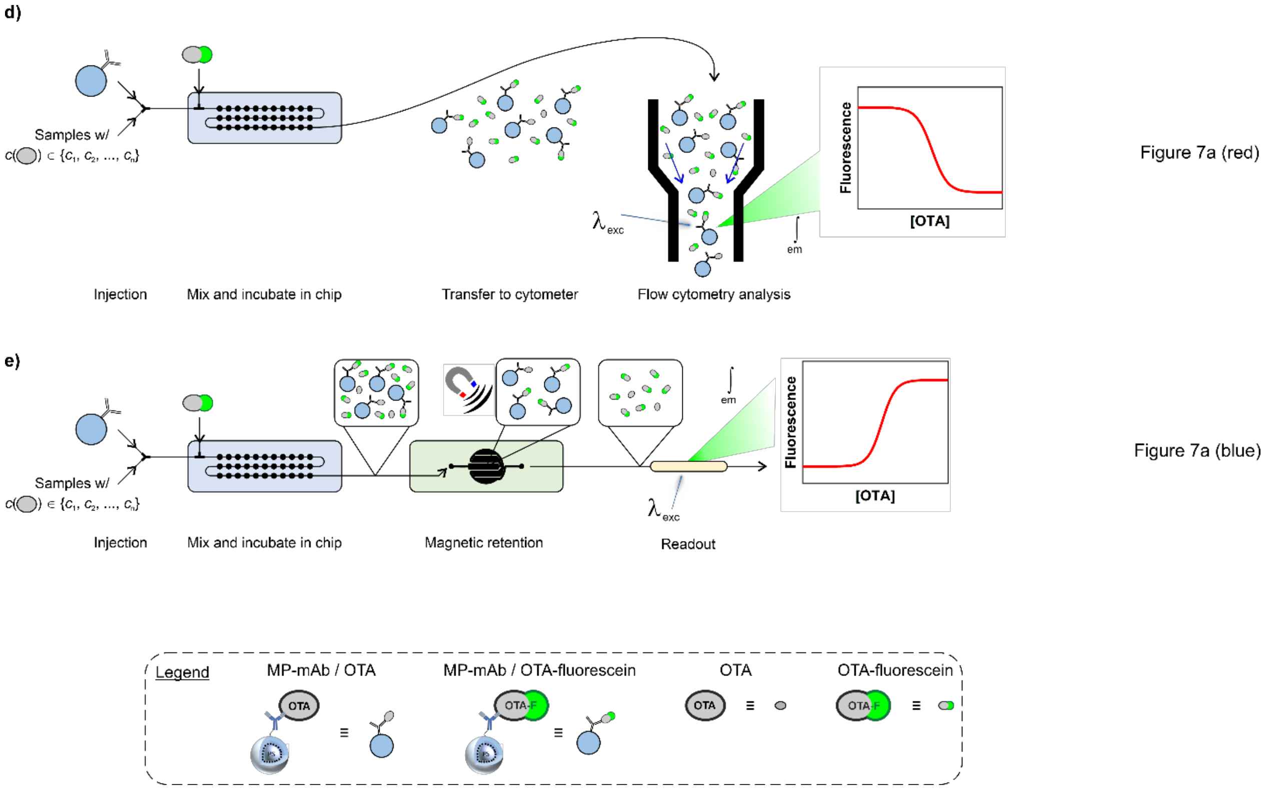

Figure S2. Schematic of assays a) using a well plate for sample processing and a flow cytometer for analysis; b) using a well plate for sample processing, a centrifugation step for particle and supernatant separation and the microfluidic detection module for analysis; c) using a well plate for sample processing, the magnetic retention module for particle capture and the detection module for analysis; d) using the mixer chip for sample processing and a flow cytometer for analysis; e) using the modular microfluidic inline platform developed in this work for sample processing and analysis.

III. Reagents and general techniques

The OTA standard and the OTA-fluorescein conjugate (OTA-F) as competitor were purchased from Aokin. The monoclonal anti-OTA antibody e#115 was previously produced and characterized.[1, 2] The preparation and characterization of the particles used here was also recently described.[3, 4] 1-Ethyl-3-[3-dimethylaminopropyl]carbodiimide hydrochloride (EDC, Merck) and *N*-hydroxy-sulfosuccinimide sodium salt (sNHS, Sigma) were used for particle activation. Phosphate buffered saline (PBS, pH 7.4, 10 mM, 130 mM NaCl), phosphate buffer (PNa, pH 7.4, 75 mM) and 2-(*N*-morpholino)ethanesulfonic acid buffer (MES, pH 6.0, 10 mM) were prepared in Milli-Q grade water. All measurements were realized in triplicates to ensure sufficient robustness of the analyses.

Cytometry measurements were performed on a BD Accuri C6 instrument recording the forward scattering (FSC) and sideward scattering (SSC) of the particles at 180° and 90° angles, respectively. Additionally, the fluorescence signal in the FL1 channel (518 nm, 533/30.H filter) was also captured. SEM images of the particles were obtained using a Zeiss Supra 40 Scanning Electron Microscope (Zeiss), equipped with a high-resolution cathode (Schottky field emitter), an Everhart-Thornley secondary electron (SE) detector, and an SE InLens detector.

Antibody selectivity was evaluated by ELISA assay in high-binding flat-bottom 96-well polystyrene Costar microtiter plates from Corning. HRP-labelled goat anti-mouse (HRP–GAM) was from BioRad Laboratories. *o*-Phenylenediamine substrate (OPD) was from Sigma. Microwells were washed with an ELx405 microplate washer and absorbance values from microplates were read with a PowerWave HT reader, both from BioTek Instruments.

Synthesis of hybrid superparamagnetic particles

The superparamagnetic microparticles were prepared in analogy to the particles in refs. [3-6] with polystyrene (PS) as the core, iron oxide (Fe_3O_4) nanoparticles as a magnetizable interlayer, and an outer silica shell for protection and further functionalization.

The synthesis of the PS particles was carried out via dispersion polymerization by reacting a solution of 170 mg of PVP10 in 10 mL of EtOH with 1 mL of styrene, filtered through basic aluminum oxide, in a glass vial after flushing the mixture with argon for 30 min and subsequently initiating it by the addition of 0.5 mL of a solution of 105 mg of ACVA in 10 mL of MeOH, flushed with argon, under stirring at 70 °C in an argon atmosphere overnight. The resulting particles were centrifuged, washed with water and EtOH multiple times, and then dried at room temperature.

In parallel, superparamagnetic iron oxide nanoparticles (SPIONs) were prepared using 0.465 g of $FeCl_3 \cdot 6H_2O$ and 0.172 g of $FeCl_2 \cdot 4H_2O$ dissolved in 100 mL of Milli-Q water in a round-bottom flask. The solution was purged with argon for 20 min before slowly adding a solution of 4 g of PVP10 in 58 mL of NH_3 solution (16%). The reaction mixture was stirred at 150 rpm for 1.5 h using a mechanical stirrer. The particles were then washed several times with water using magnetic separation and stored in a refrigerator at a concentration of approximately 3% (w/v) in Milli-Q water.

Third, the PS cores were coated with a layer of SPIONs by suspending 60 mg of PS cores and 2 mL of Fe_3O_4 particles (3% in water) in 30 mL of Milli-Q water in Falcon tubes. The coating process was carried out by placing the tubes on a rotator plate at 40 rpm for 1.5 h. Afterward, the SPION@PS particles were washed twice with water and once with ethanol, using magnetic separation, before drying.

Fourth, coating of the particles with a silica shell was achieved by adding 555 µL of a NH_3 solution (32%) to a dispersion of 60 mg of SPION@PS particles in 30 mL of EtOH and 1 mL of Milli-Q water while stirring at 150 rpm using a mechanical stirrer. Then, 555 µL of TEOS was added dropwise. The mixture was stirred overnight at 38°C, followed by multiple washes with water and EtOH, using magnetic separation. Finally, the particles were dried at room temperature.

Figure S3 collects the cytometer measurements performed to control the synthesis procedure as discussed in detail in refs. [3, 4].

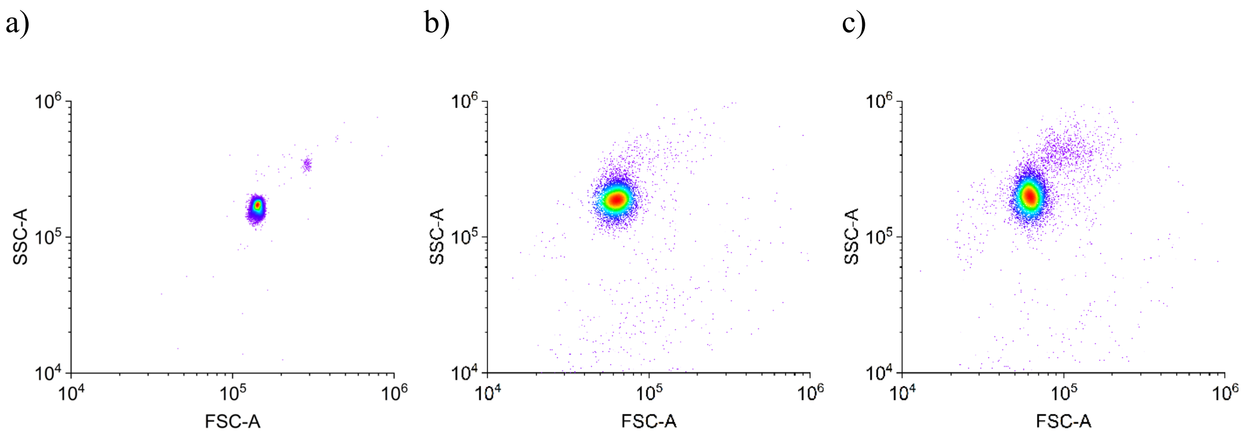

Figure S3. Dot plots of side vs forward scattered signals recorded in SSC and FSC channels showing the dispersity of a) the PS particles, b) the SPION@PS particles and c) the silica-coated SPION@PS particles.

Functionalization with amino groups

According to a previously reported protocol,[7] the silica-coated SPION@PS particles were functionalized with amino groups by first activating the particles' surface after suspending 20 mg of the particles in 800 µL of EtOH, adding 400 µL of 1 M HCl in EtOH, and sonicating the mixture in a sonication bath for 5 min. Afterward, the particles were washed twice with 400 µL of EtOH and redispersed in 400 µL of EtOH. For amino modification, 8 µL of APTES was added to the particle dispersion, and the mixture was allowed to react in a thermomixer (800 rpm) at 40°C

overnight. Subsequently, the particles were washed three times with a mixture of EtOH:H_2O in a 1:1 ratio, before drying in vacuum at room temperature.

Functionalization with carboxylic acid groups

To modify the surface of the materials with carboxylic acid groups for facile biomolecule attachment, 5 mg of the corresponding amino-modified particles was dispersed in 1.5 mL of absolute EtOH in a 2 mL Eppendorf tube. A solution of 30 μL 10% w/v of succinic anhydride in dimethylformamide was added to the particle dispersion, and the mixture was then allowed to react in a thermomixer (800 rpm) at 40°C overnight. Afterward, the particles were washed three times with a mixture of EtOH:H_2O in a 1:1 ratio. In the final step, 500 μL of ethanol was added to the particles to obtain a stock solution with a final concentration of 1% (w/v).

Figure S4 shows a cytometric analysis of the final beads after antibody immobilization as described in the Experimental Section of the main text.

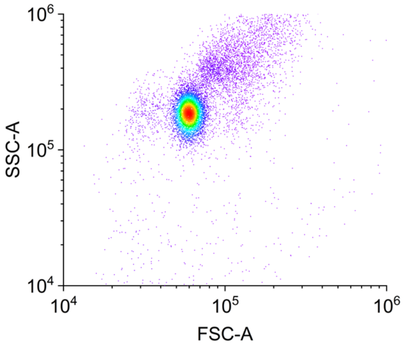

Figure S4. Dot plot of SSC vs FSC of the mAb-coated particles.

IV. Antibody cross-reactivity

The cross-reactivity of the selected antibody was evaluated by ELISA in a competitive indirect assay using the homologous conjugate as competitor.[1] Antibody OTAe#115 and competitor OVA-OTAe were used at 0.1 mg L^{-1}. A 96-well plate was coated overnight at room temperature using 100 μL of OVA-OTAe solution in 50 mM carbonate buffer, pH 9.6. Afterwards, the microwells were washed and the competitive reaction was performed by mixing 50 μL of toxin standard solution in PBS and 50 μL of antibody solution in PBS containing 0.05% (v/v) Tween 20 (PBS-T) per well. After 1 h at room temperature and a washing step, the immunochemical reaction was amplified by adding 100 μL of 5000-fold diluted GAM–HRP (Goat anti-Mouse-HRP conjugate) in PBS-T per well. After a 1 h incubation at room temperature, the retained peroxidase activity was revealed with 100 μL of enzyme substrate solution (2.5 g L^{-1} of OPD and 0.012% (v/v) of H_2O_2 solution in 25 mM citrate and 62 mM phosphate buffer, pH 5.4) per well and incubating 10 min at room temperature. Finally, the enzyme activity was stopped with 100 μL of 1 M H_2SO_4 per well. The absorbance was read at 492 nm using a reference wavelength of 650 nm. Nine mycotoxins were evaluated as structurally and application-relevant interferents. Figure S5 shows that except for the structurally very closely related analytes of the ochratoxin family, OTB and OTC, all the application-relevant mycotoxins show no interference.

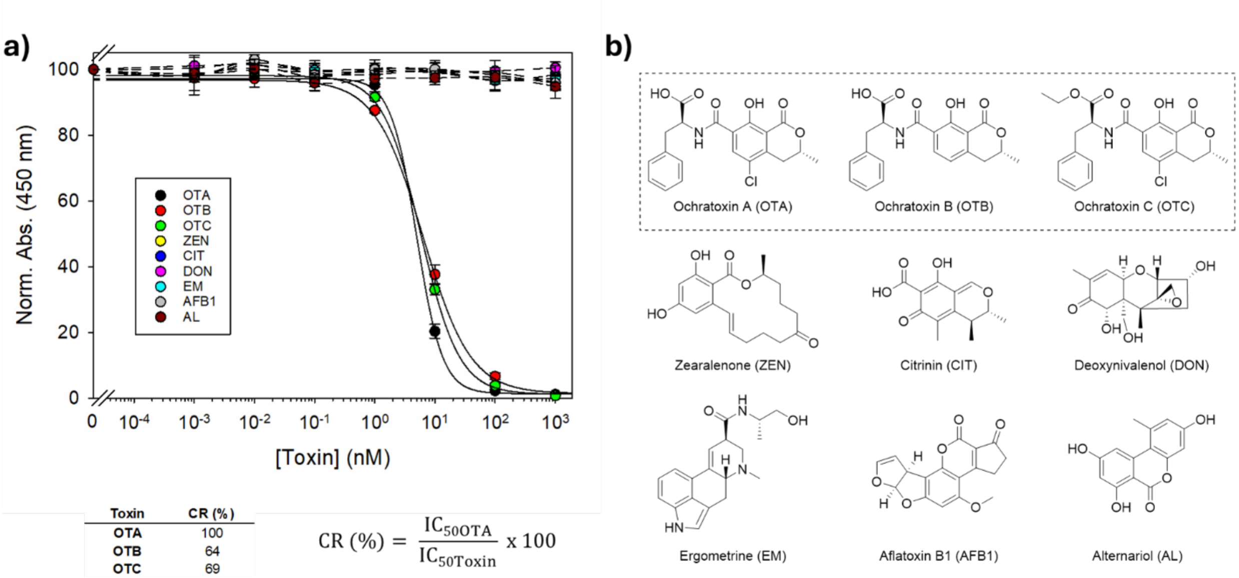

Toxin	CR (%)
OTA	100
OTB	64
OTC	69

$$\text{CR (\%)} = \frac{\text{IC}_{50\text{OTA}}}{\text{IC}_{50\text{Toxin}}} \text{ x } 100$$

Figure S5. a) Set of calibration curves obtained for the nine tested mycotoxins and cross-reactivity (CR) calculations for the two recognized toxins: OTB and OTC. b) Chemical structures of the nine mycotoxins.

V. Calibration curves

The calibration curves were constructed from the concentration-dependent measurements as described in detail in the main text. For curves without the pearl chain mixer, a 4-parameter logistic (4-PL) model was employed for data fitting (Eq. S1), in accordance with the binding behavior of biological binders.

$$y = \frac{A - D}{1 + \left(\frac{x}{C}\right)^B} + D$$

Equation S1. 4-Parameter logistic model used to describe the assay's sigmoidal dose-response curve obtained with the developed system and well plate-based mixing and incubation, as often seen in receptor-ligand binding assays: A is the infinite-dose asymptote; B is the Hill's slope parameter; C is the point of inflection (IC_{50}); D is the zero-dose asymptote. The IC_{50} value is directly corresponding to the C parameter. The limit of detection (LoD) corresponds to the IC_{10} value, calculated as 10% of inhibition for cytometer assays and 10% of response for microfluidic assays.

For the calibration curves built with signals coming from the setup using the pearl chain passive mixer, a 5-parameter logistic (5-PL) model was employed for data fitting (Eq. S2).

$$y = \frac{A - D}{\left(1 + \left(\frac{x}{C}\right)^B\right)^E} + D$$

Equation S2. 5-Parameter logistic model used to describe the assay's sigmoidal dose-response curve obtained with the developed system and pearl chain mixing and incubation, as often seen in asymmetrically responding receptor-ligand binding assays: A is the infinite-dose asymptote; B is the Hill's slope parameter; C is the point of inflection (IC_{50}); D is the zero-dose asymptote; E is the asymmetry parameter. The IC_{50} value is directly corresponding to the C parameter. The limit of detection (LoD) corresponds to the IC_{10} value, determined graphically as 10% of inhibition for cytometer assays and 10% of response for microfluidic assays.

A 5-PL model is relevant for cases where some asymmetry is observed in the calibration curve, and a 4-PL model does not fit the results with acceptable goodness, especially at high analyte concentrations. This behavior has been previously observed by other groups using particles in

microfluidic channels.[8] It arises from the mixing mechanism within the pearl chain passive mixer where successive convergent–divergent cross-sections induce the formation of vortices that break down the two incoming solutions into layers or plugs of smaller sizes.[9] The resulting more heterogeneous mixing compared to well plate mixing probably influences the capture of the analyte and the competitor by the antibodies, thereby slightly modifying the competition results.[10] This is especially evident for high OTA concentrations, for which asymmetry is observed.

To facilitate comparison between the single units, Table S1 combines the assay performance data of the various setup and instrument combinations used in this work. Taking the IC_{50} data as a measure, not surprisingly and presumably based on the detection power (lasers, photomultiplier tubes), the cytometer was more sensitive than the detection chip, cf. #1 *vs* #2 and #4 *vs* #5. In contrast, in our study, the pearl chain mixing/incubation chip performed better than the well plate-based mixing and incubation when directly followed by particle detection in the cytometer, cf. #4 *vs* #1. However, when used together with the magnetic retention and detection chips, pearl chain and well plate incubation showed similar performance, cf. #5 and #3. In addition, the combination of mixing and retention chips performed better than well plate mixing and centrifugation, cf. #5 *vs* #2. In conclusion, the pearl chain mixer and magnetic retention modules perform excellently compared to standard lab equipment (well plate incubation, centrifugation), while the main difference in sensitivity stems from the detection unit and scales with the excitation and detection power of the instrument, with the cytometer generally outperforming the detection chip. However, this was to be expected and scales with complexity and price while clearly being disadvantageous in terms of onsite capability.

Table S1. Fitting results using 4-PL and 5-PL models for the calibration plots in Figures 4, 6 and 7

No.	Figures	Mixing/ incubation	Particle separation	Detection	Mean standard deviation (3σ)	Model	r^2	IC_{50}	IC_{10} (LoD)
					%			µg L^{-1}	µg L^{-1}
#1	4, S2a	Well plate	None	Cytometer	11.5	4-PL	0.997	5.7 ± 1.3	0.1 ± 0.1
#2	4, S2c	Well plate	Centrifuge	Chip	3.0	4-PL	0.995	24.3 ± 7.2	1.0 ± 0.5
#3	6, S2d	Well plate	Magnet	Chip	6.1	4-PL	0.992	12.9 ± 4.7	0.2 ± 0.2
#4	7a, S2f	Pearl chain	None	Cytometer	9.4	5-PL	0.998	0.7 ± 0.2	0.2 ± 0.2
#5	7a, S2b	Pearl chain	Magnet	Chip	9.2	5-PL	0.998	10.3 ± 1.6	1.2 ± 0.9

Representative raw data of calibration measurements obtained with the flow cytometer are given in Figure S6. Representative raw data obtained with the inline detection module are provided in Figure S11 below.

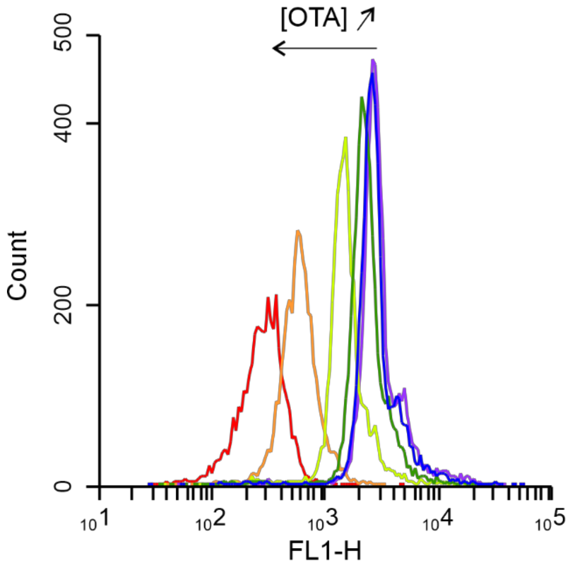

Figure S6. Histogram of emission intensities in FL1-H channel from the flow cytometry measurements for OTA in water samples after competitive assay in offline well plate-based sample processing (corresponding to the calibration plots in Figure 4).

VI. Cytometric control measurements

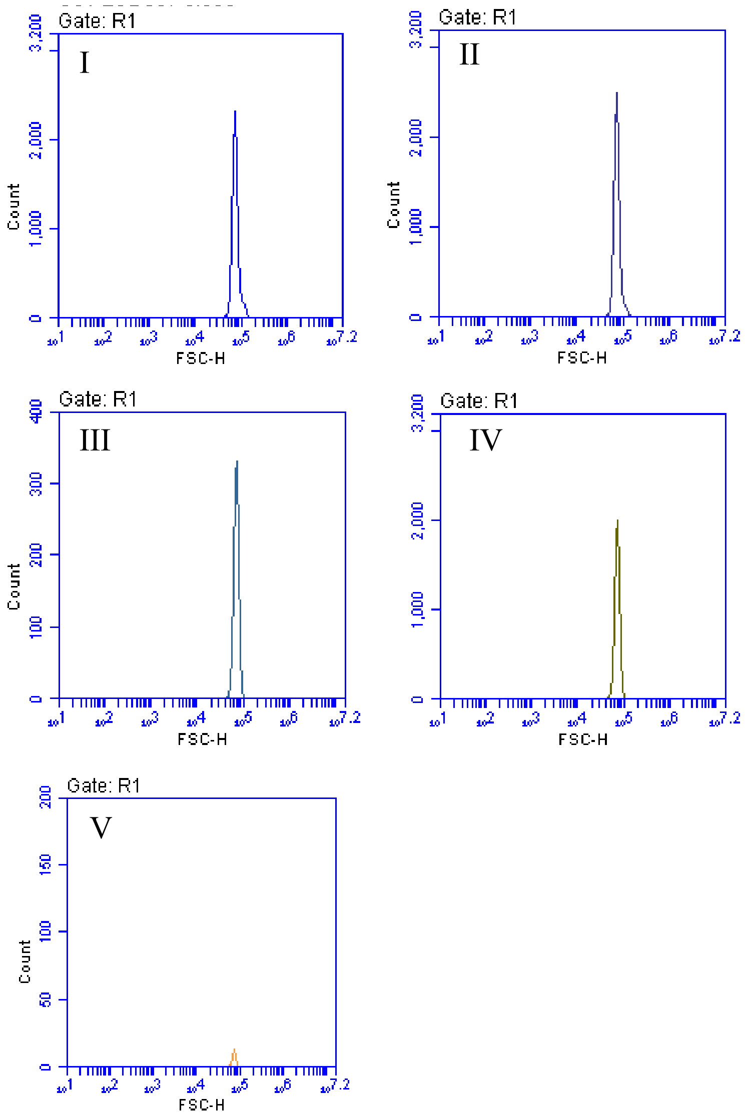

	Particle count
I	8090
II	8779
III	1106
IV	7374
V	28

Figure S7. (I) Particle control count without going through the PDMS chip, (II) Particle count after the PDMS chip without magnet, (III) Particle count after the PDMS chip with magnet, (IV) Particle count after removing the magnet and washing; (V) Particle count after washing.

VII. Performance of different magnets in retention module

Table S2. Combinations of magnet types and chips tested via the injection of 100 µL of particle suspension (0.05% w/v) and two successive washes after removal of the magnet.

Magnet type	M	L	XL	Grate[11]	M’[12]
Picture	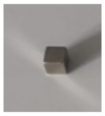	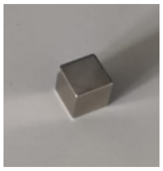	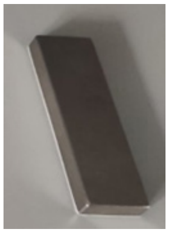	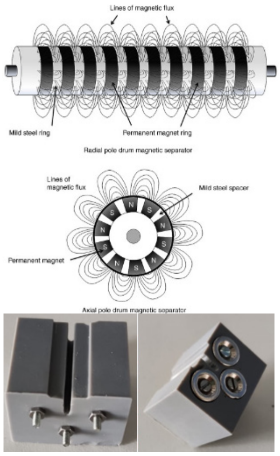	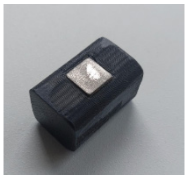
Shape & size	Cubic<br>5 mm	Cubic<br>10 mm	Rectangular<br>2×20×50 mm	Tri-cylindrical<br>*l* = 27 mm	Cubic<br>8 mm
Chip type	Tubing				PDMS
Cytometer control	Event per µL				
Magnet	1666	909	286	224	40
Wash 1	1666	556	172	39	1110
Wash 2	54	91	52	13	0
Retention efficiency	51%	42%	44%	19%	97%

IX. Indirect quantification of OTA through quantification of OTA-F bound by particles

As the inline setup allows to capture the magnetic particles, two indirect detection approaches should principally also be possible to indirectly quantify OTA, which might be advantageous to run internal controls or be closer to expectations of users that are commonly utilizing conventional competitive immunoassays, i.e., measuring fluorescence of the immobilized particles in the capture area (Figure S8a) or releasing the particle after each supernatant measurement by removal of the magnet, washing them out of the retention module, collecting and analyzing the fractions in a cytometer, see Figure S8b. However, both approaches have serious practical drawbacks and do not yield data of acceptable quality. In view of the first approach, the beads could not be captured in a monolayer in the capture area, i.e., on the glass substrate at the bottom of the chip through which the magnet operates, with all the fluorescent entities pointing to the solution side so that they could be reliably fluorometrically interrogated in the capture area for quantification. For the second approach, calibration curves that are comparable in quality to those shown in Figure 6 could not be obtained. The reason in both cases is that, as confirmed by microscope image analysis of the area in which the magnet captured the particles in the chip, the particles cannot be captured in a homogeneous and defined manner (Figure S9a) so that also too high flow rates of the washing solution (>200 µL min^{-1}) are necessary to quantitatively release the beads instantaneously (Figure S9b), yielding suspensions that are transiting too quickly in the detection module for reliable analysis. (Additional steps to reduce the amount of liquid were not attempted, introducing further errors.) Furthermore, a disadvantage for both approaches is that they would require discontinuous operation of the assay/device within a single run, increasing assay times and potentially introducing additional measurement uncertainties.

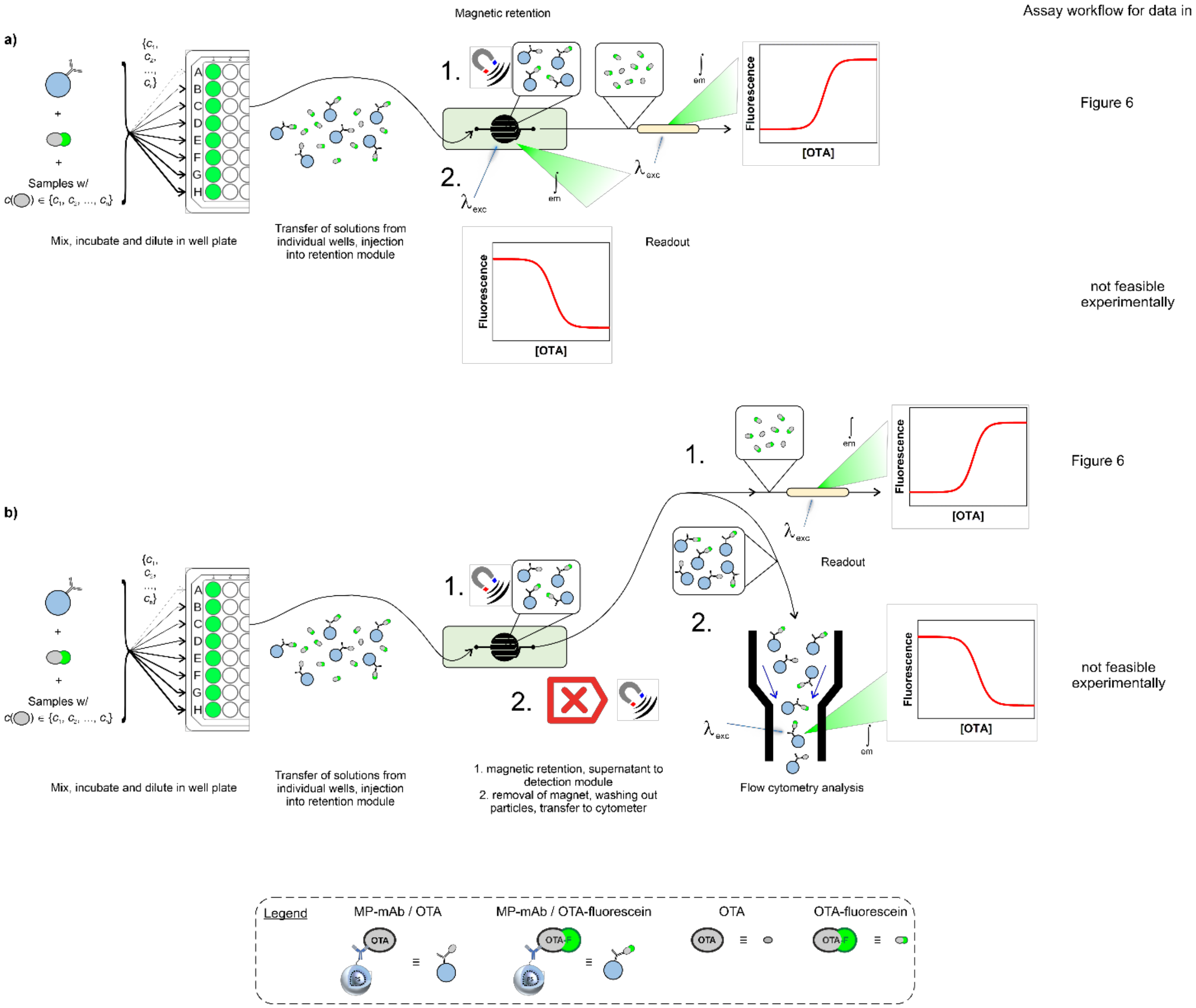

Figure S8. Schematic of hypothetical tandem assays quantifying bound OTA-F directly and indirectly a) using a well plate for sample processing, then a hypothetical magnetic retention and detection module, first for particle capture and second for analysis of particle-bound OTA-F, and the detection module for free OTA-F analysis; b) using a well plate for sample processing, then first the magnetic retention module for particle capture and the detection module for analysis and second removing the magnet, washing out the particles and transferring them to the cytometer for analysis.

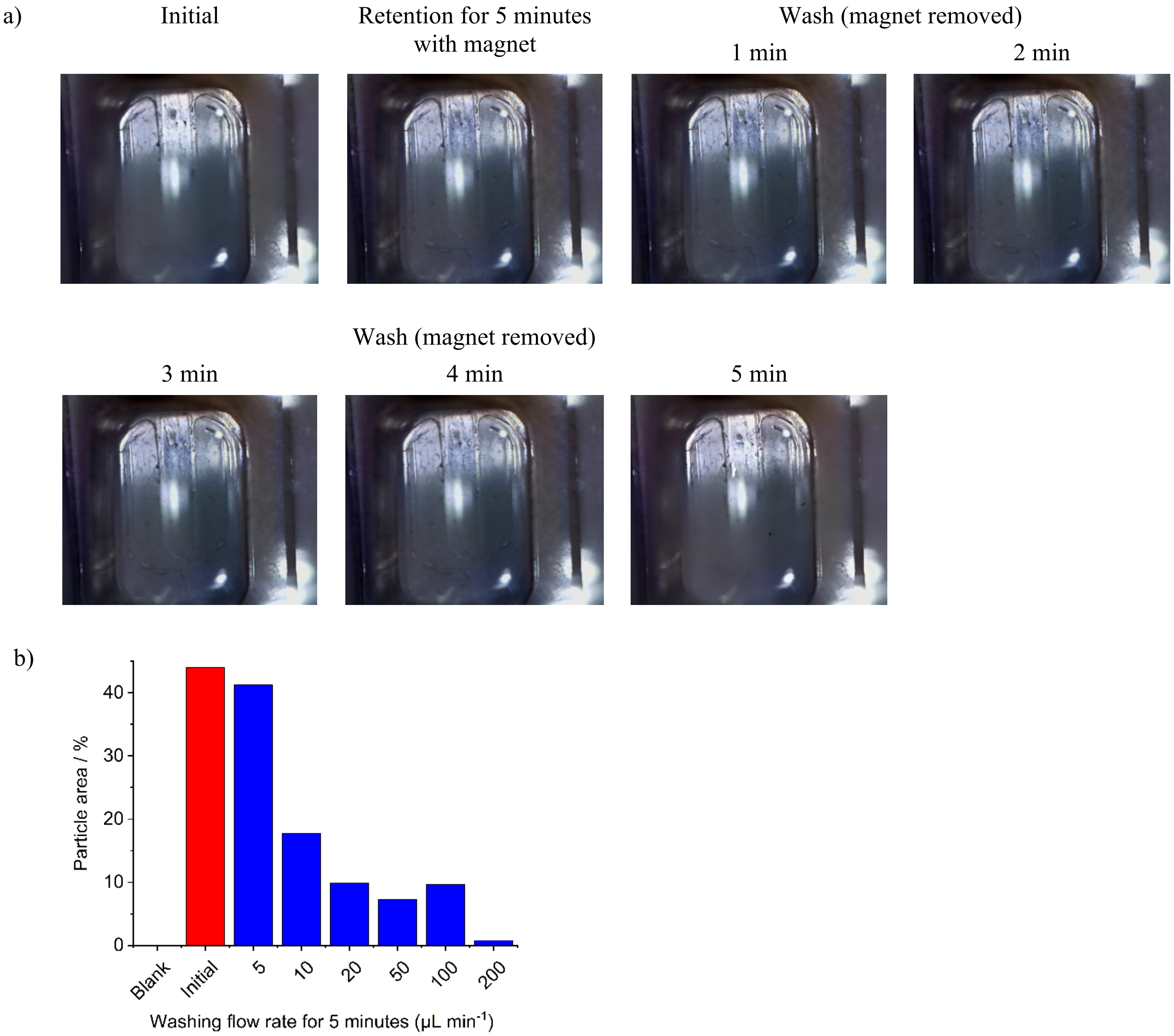

Figure S9. a) Representative microscope images of the capture area of the retention module's capture chamber, showing the inhomogeneous retention of the particles by the magnet, and slow release of the particles upon removal of the magnet. b) Percentage of the capture area covered by particles as extracted from the microscope images before and after magnet removal and 5 min washing with PNa buffer. Even at high flow rates, a sizeable number of mAb-particles remain in the retention chamber, making it impossible to run direct indirect control experiments with the inline setup.

X. Incubation time

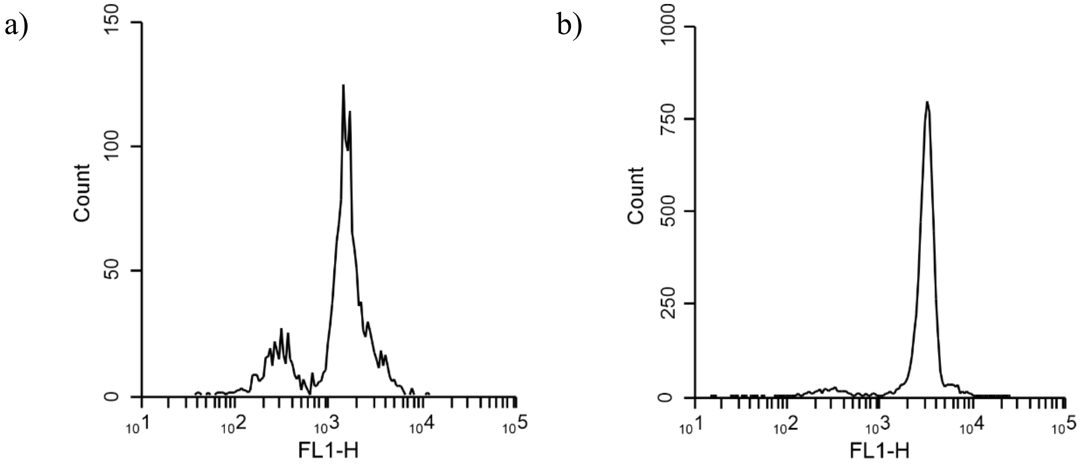

Figure S10. Fluorescence histograms (FL1-H channel of cytometer) of particle suspensions after different incubation times via different transit times in pearl chain mixer at (a) 1.8 µL min^{-1} of mAb-particles (0.05%), 0.6 µL min^{-1} of OTA-F (0.5 µM) and 9.6 µL min^{-1} of PNa buffer (75 mM) as the sample, yielding a total incubation time of 10 min, and (b) 3.6 µL min^{-1} of mAb-particles (0.05%), 1.2 µL min^{-1} of OTA-F (0.5 µM) and 19.2 µL min^{-1} of PNa buffer (75 mM) as the sample, yielding a total incubation time of 5 min.

The optimal incubation time required for the competitive reaction was determined using flow cytometry (Figure S10), by evaluating both signal intensity and particle population distribution under different incubation conditions. This evaluation was performed after mixing the three components in the reaction module, which was tested without the other modules attached to it to ensure controlled and reproducible conditions during the optimization process. As Figure S10 shows, longer incubation times such as 10 min were not optimal as two distinct particle populations were found in flow cytometry and the overall signal intensity was lower compared to 5 min. After finding the optimum incubation times at ≤5 min with this workflow, the other modules were added to the mixer module and the overall performance was assessed. These experiments revealed that

fast incubation times such as 3 min were too fast for efficient particle capture in the magnetic retention unit. Therefore, 5 min of residence time in the mixer module was selected for the final assay.

If one calculates the residence times t_r in the three modules via the known internal volumes and the total flow rate, t_r is determined to 5 min for the mixing/incubation module, to 30 s for the retention module and to 30 s in the detection module, which consists of a simple tubing section.

XI. Laboratory prototype setup

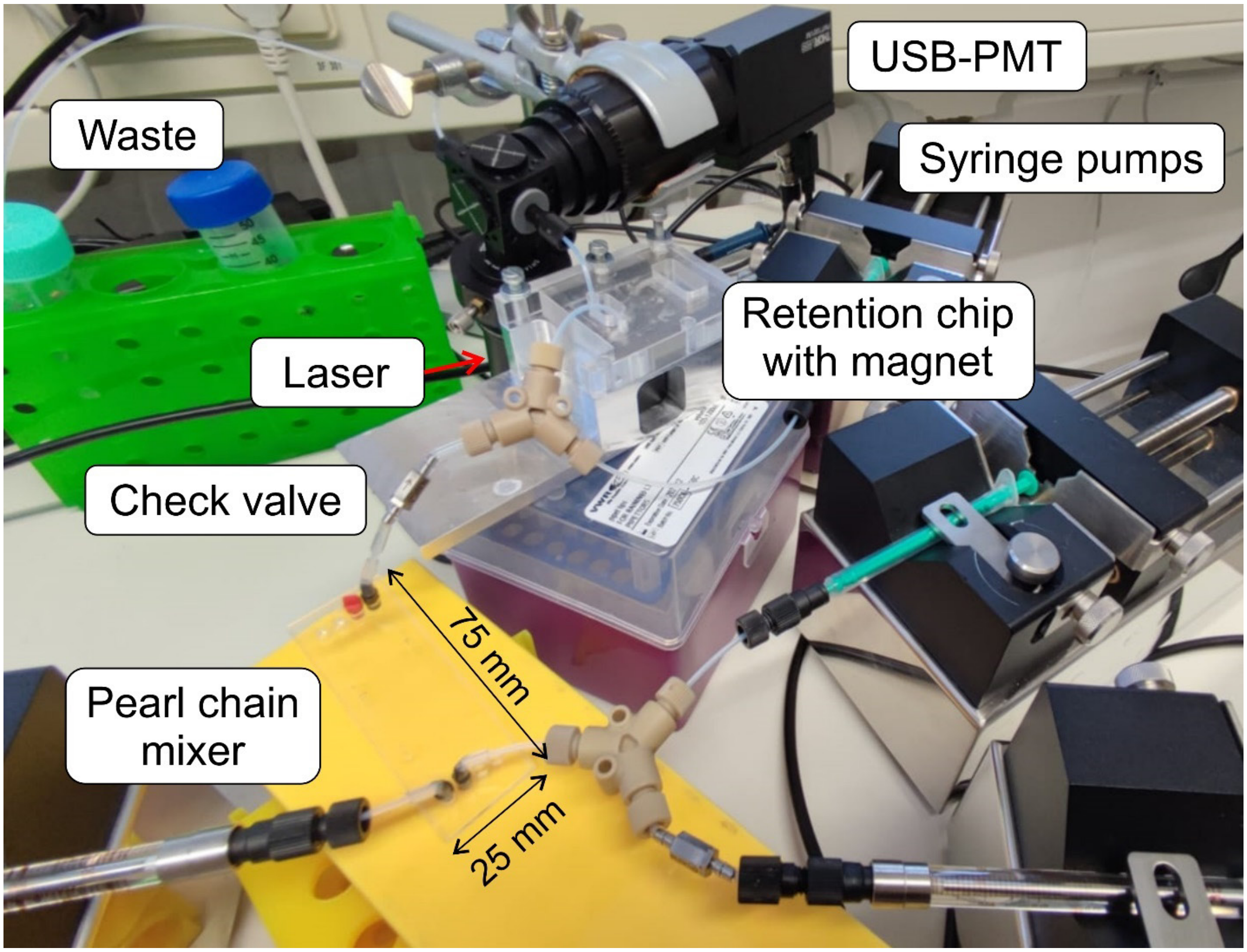

Figure S11. Photograph of laboratory prototype of the assay setup. The single components are indicated in the image; the microscope slide (75×25 mm) of the pearl chain mixer serves as a scale. For signal transduction, the setup was built with stable optomechanical parts to hold all the required lenses and filter, the excitation source is a fluorescein matching 488 nm USB laser module, the detector is a USB photomultiplier connected to a USB oscilloscope that converts the optical signal into a digital signal for readout. It should be noted that a final device can be significantly smaller using dedicated miniaturized and automated syringe or piezo pumps, together with the replacement of the hardware for signal conversion (USB oscilloscope and computer not shown on the picture) by a miniaturized microcontroller driven through a smartphone.[13]

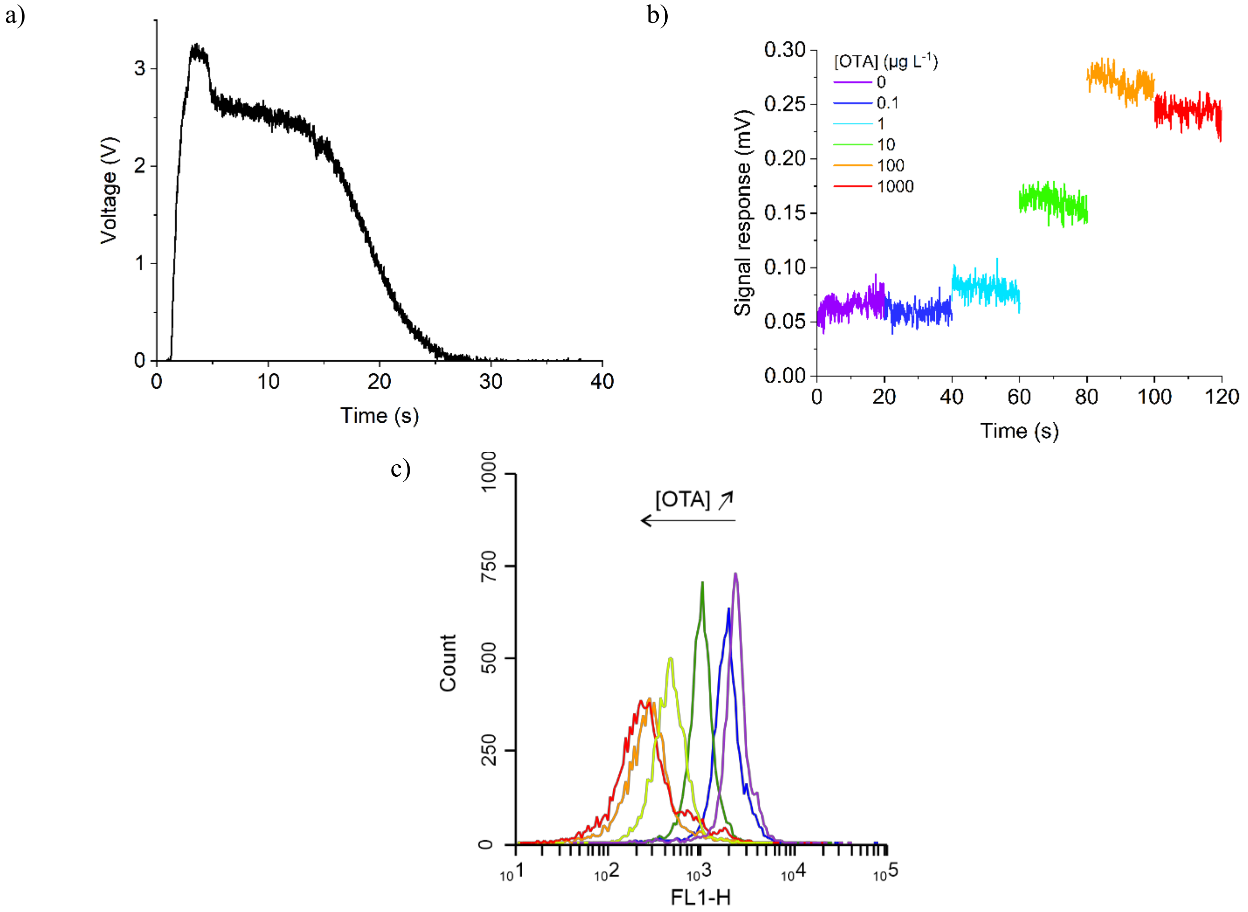

Figure S12. a) Raw signal recorded upon injection of 5 nM of an aqueous solution of OTA-F into the microfluidic system with high PMT gain; b) raw signals obtained with the complete setup upon increasing concentrations of OTA and c) raw signals obtained with cytometric offline detection. To obtain the plot shown in Figure 7, raw signals were integrated for 20 s corresponding to the transit time of the highest concentration of OTA-F in front of the detector as seen on the response curve in a).

XII. Wheat flour sample extraction and measurement

For sample extraction, a standard protocol for mycotoxin extraction from wheat flour samples was followed. Such protocol relies on the acidic properties of OTA, utilizing an initial extraction with an acidic organic solvent for the separation of OTA from polar, water-soluble matrix compounds. This was followed by a second extraction using a basic buffered solution, which allowed the recovery of the toxin in an aqueous phase compatible with the antibody-based detection system. The protocol can be run with limited equipment, for example onsite, at a mill. The overall extraction procedure is relatively quick, requiring only 15–20 minutes, primarily due to centrifugation steps.

Detailed procedure:

A wheat flour sample containing OTA at 0.2 µg kg^{-1}, the concentration known through a regular analysis by Eurofins commissioned by the mill and confirmed internally by BAM's Organic Trace and Food Analysis Division, was used for the recovery study. Two portions of 5 g of the sample were spiked at 5 and 20 µg kg^{-1} using a concentrated stock solution of OTA in methanol. After overnight drying, both spiked samples and a blank were suspended in 20 mL of CH_2Cl_2 in a 50 mL tube along with 500 µL of H_3PO_4 (6 M). The tube was placed on a rotator plate for 15 min at 40 rpm and then centrifuged at 8000 rpm for 10 min. From the resulting mixture, 1 mL of the organic phase was transferred to a 2-mL Eppendorf tube and 250 µL of bicarbonate buffer (50 mM, pH 9.6) was added. The Eppendorf tube was mixed on a rotator plate for 10 min and then centrifuged at 6000 rpm for 3 min, and the aqueous phase was collected. Finally, the extract was diluted in phosphate buffer 100 mM pH 7.4 using a dilution factor of 1/10.

a)

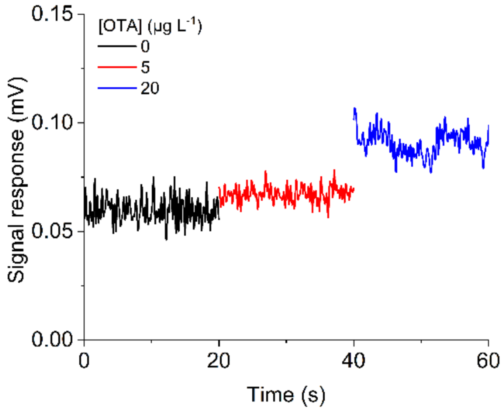

b)

Sample	# 1	# 2
Spiked concentration (μg L^{-1})	5	20
Found concentration (μg L^{-1})	3.8 ± 0.3	17 ± 1.5
Recovery (%)	76	85

Figure S13. a) Raw signals obtained from assays with the complete setup for OTA spiked flour samples and b) respective calculated concentrations and recoveries.

XIII. Method comparison

Table S3. Comparison with representative conventional methods from the literature.

#	Method[a]	Mechanism	LoD buffer (µg L^{-1})	Range[b] (µg L^{-1})	Assay time (min)	LoQ sample[c] (µg L^{-1})	Commodity	Sample treatment	Regen-eration[d]	Onsite capability	Multianalyte potential[e]	Price per sample	Ref.
1	HPLC-FLR	Fluorescence detection after LC separation	0.25	0.05–1	40	1.5	Wheat flour	Extraction + immunoaffinity column	Yes	No	Related structures	≈ 400€	[14]
2	LC-MS/MS	Mass spectrometry detection after LC separation	0.12	0.2–4.8	90	0.6	Wheat/bread	Extraction + evaporation	Yes	No	Yes	≈ 400€	[15]
3	ELISA	Competitive immunoassay with colorimetric detection	0.01	0.005–0.2	90	0.5	Wine	Polymer clarification	No	Yes	No	≈ 10€	[2]

Table S4. Comparison with representative miniaturized methods from the literature.

#	Method[a]	Instrumentation	Mechanism	LoD buffer (µg L^{-1})	Range[b] (µg L^{-1})	Assay time (min)	LoQ sample[c] (µg L^{-1})	Commodity	Sample treatment	Regen-eration[d]	Onsite capability	Multianalyte potential[e]	Ref.
4	Electrochemical immunoassay	Modified electrodes + electrochemical workstation + discontinuous workflow	Analyte-mediated modulation of redox marker access to electrode surface	0.08	0.1–1.0	40	n.r.	Malt	Extraction	No	No	Different structures (limited)	[16]
5	Fluorescent LFIA	Test strip + fluorescence analyzer for strips	Immunochromato-graphic test strips with fluorescence detection	0.07	0.1–10	30	0.5	Corn flour	Extraction	No	Yes	Different structures (limited)	[17]
6	Colorimetric test	Strip + holder + smartphone	Dye displacement from protein complex	0.4	0.12–20	40	0.5	Flour	Extraction	No	Yes	No	[18]
7	Colorimetric (HRP/TMB) capillary chip immunoassay	Capillary multicomponent chip + smartphone	Competitive immunoassay with surface-bound antigens and HRP-labeled antibodies	<40	25–100 (semi-quantita-tive)	15	n.r.	Corn	Extraction	No	Yes	Different structures	[19]
8	PEC biosensor	Modified electrodes + electrochemical workstation + LED lamp	Aptamer recognition inhibits photocurrent decoloration	0.3	1–250	n.r.[f]	1	Corn	n.r.	No	(Yes)	Different structures (limited)	[20]
9	Visual / photoelectro-chemical sensing	Modified electrodes + electrochemical workstation + Xe lamp	Aptamer recognition inhibits photocurrent color formation by oxidation	0.01	0.002–300	60	0.1	Corn flour	n.r.	No	(Yes)	Different structures (limited)	[21]
10	OFF–ON ECL sensor	Modified electrodes + ECL workstation	Aptamer recognition releases ECL quencher	0.03	0.1–320	100	5	Wheat + oat	Extraction	No	Yes	Different structures (limited)	[22]

#	Method[a]	Instrumentation	Mechanism	LoD buffer (µg L^{-1})	Range[b] (µg L^{-1})	Assay time (min)	LoQ sample[c] (µg L^{-1})	Commodity	Sample treatment	Regen-eration[d]	Onsite capability	Multianalyte potential[e]	Ref.
11	White light reflectance spectroscopy immunosensor	Biochip + analyzer	Competitive immunoassay with mass deposition for reflectance detection	0.03	0.03–200	30 + 30	0.06	Corn + wheat fluor	Extraction	12 times	Yes	Different structures (limited)	[23]
12	Microarray-based FIA	Multicomponent chip + protein microarray + analyzer	Competitive immunoassay with fluorescence detection	1.2	2–20	50	12	Corn	Extraction	No[g]	Yes	Different structures	[24]
13	Multiplexed microfluidic FIA	Microfluidic device + immobilized beads + laser diode + photodiodes	Competitive immunoassay with fluorescence detection	0.7	0.5–50	20	3	Corn	Extraction	No	Yes	Different structures (limited)	[25]
14	Microfluidic FIA	Multicomponent chip + analyzer	Aptamer recognition releases fluorescent dye	1.3	5–200	5	3.9	Wheat + beer	Extraction	No	Yes	Different structures (limited)	[26]
15	Bead-based microfluidic colorimetric (HRP/TMB) immunoassay	Flow cell + screen-printed electrode + magnet + smartphone	Competitive immunoassay with surface-bound antibodies and HRP-labeled OTA	0.06	0.5–5	60	0.5	Beer	Dilution (matrix dependent LoD)	No	Yes	Different structures (limited)	[27]
16	CL chip-based LFIA	Multicomponent cartridge + holder + smartphone	Competitive immunoassay with CL detection	0.1	up to 25	45	n.r.	Wine + coffee	Extraction	No	Yes	Different structures (limited)	[28]
17	Bead-based microfluidic FIA	Modular microfluidic setup	Competitive immunoassay with fluorescence detection	0.8	0.1–1000	10	5	Wheat flour	Double extraction	Yes	Yes	Different structures	This work

For footnotes, see next page.

[a] (HP)LC = (high-performance) liquid chromatography, FLR = fluorescence, MS = mass spectrometry, ELISA = enzyme-linked immunosorbent assay, LFIA = lateral flow immunoassay, PEC = photoelectrochromic, (E)CL = (electro)chemiluminescence, FIA = fluoroimmunoassay, HRP/TMB = horseradish peroxidase/3,3′,5,5′-tetramethylbenzidine, icFLISA = indirect competitive fluorescence linked immunosorbent assay.

[b] Deduced from the respective plots in the publications.

[c] Limit of quantification (LoQ) in sample expressed as the lowest concentration of OTA determined.

[d] Regeneration of the instrument/device for successive measurements of multiple samples.

[e] Related structures = only analytes of similar chemical classes can be detected within a single run; different structures = analytes of different chemical classes (acids, bases, neutral) can be principally detected within a single run if binders are available; limited = because of their simplicity and the inherent limitations of separation and read-out, strips possess only a limited multiplexing potential; chips with surface-bound binders also have limitations because of spatial restrictions; ECL and PEC approaches have limitations due to the availability of chromophores and spatial constraints.

[f] Not reported.

[g] Parallel analysis of samples defined by microarray.

XIV. Measurement uncertainties

Because of the multiplicative and quotient forms of the respective equations, and because correlations between the quantities are assumed to be negligible, summation of the squares of the relative uncertainties was performed.[29, 30]

Preparation of antibody-coated particle suspension and OTA-F competition solution:

Pipetting of 20 µL of the antibody stock solution (Eppendorf Reference pipette 100 µL ± 0.4 µL); u_{rel}^{d1} = 2 %

Dispersing in 200 µL of fresh PBS (Eppendorf Reference pipette 1000 µL ± 3 µL); $u_{rel}^{d2} = 1.5$ %

Dilution of commercial 1 µM solution of OTA-F in 200 µL of fresh PBS (Eppendorf Reference pipette 1000 µL ± 3 µL); $u_{rel}^{d3} = 1.5$ %

Preparation of the OTA solutions for calibration:

Weighting of 2 mg of OTA (balance Mettler Toledo ± 0.01 mg); $u_{rel}^{w1} = 0.5\%$

Dissolving in 1.9 mL of EtOH (Eppendorf Reference pipette 5 mL ± 0.03 mL); $u_{rel}^{d4} = 1.5\%$

Pipetting of 77 µL OTA stock solution (Eppendorf Reference pipette 100 µL ± 0.4 µL); $u_{rel}^{d5} = 0.5\%$

Dissolving in 1.93 mL of EtOH (Eppendorf Reference pipette 5 mL ± 0.03 mL); $u_{rel}^{d6} = 1.5\%$

Successive dilutions in 200 µL of fresh PBS (Eppendorf Reference pipette 1000 µL ± 3 µL); $u_{rel}^{d7} = 1.5\%$

Preparation of the flour samples:

Weighting of 5 g of flour samples (balance Mettler Toledo ± 0.01 g); $u_{rel}^{w2} = 0.2\%$

Spiking with 2.5 or 10 µL of a 10 mg/L OTA solution for calibration (Eppendorf Reference pipette 10 µL ± 0.025 µL); $u_{rel}^{sp} = 0.25 - 1\%$

Dispersing in 20 mL of CH_2Cl_2 (Eppendorf Reference pipette 10 mL ± 0.06 mL); $2 \times u_{rel}^{d8} = 2 \times 0.6\%$ (n = 2)

Pipetting 1 mL of the organic phase (Eppendorf Reference pipette 1000 µL ± 3 µL); $u_{rel}^{d9} = 0.3\ \%$

Pipetting 250 µL of the bicarbonate buffer (Eppendorf Reference pipette 1000 µL ± 3 µL); $u_{rel}^{d10} = 1.2\%$

Pipetting 2.25 mL diluted in phosphate buffer (Eppendorf Reference pipette 5 mL ± 0.03 mL); $u_{rel}^{d11} = 1.3\%$

Cytometry assay:

Pipetting of solutions into the well plate; $u_{rel}^{d12} = 0.5\ \%$

Autosampler volume error (not specified); $u_{rel}^{as} = 0.5\ \%$ (assumed)

Cytometer MFI error (not specified); $u_{rel}^{cy} = 1\ \%$ (assumed)

Inline assay:

Flow rate accuracy from syringe pump; $3 \times u_{rel}^{p} = 3 \times 0.5\ \%$ (n = 3)

Output power stability of laser: $u_{rel}^{l} = 1.0\ \%$

Analysis error from the analogue to digital conversion of the PMT signal (dark noise 10 nA) by the oscilloscope; $u_{rel}^{sig} = 0.1\ \%$

Repetition of measurements; $u_{rel}^{r} = 0.4 - 8\%$

Total relative uncertainties:

$u_{rel}^{tot} = \sqrt{\sum_{all\ x} n \times {u_{rel}^{x}}^2}$ = 4.6 – 9.2% (Figure 1a); 4.7 – 9.2% (Figure 1b)

XV. References

(1) López-Puertollano, D.; Mercader, J. V.; Agullo, C.; Abad-Somovilla, A.; Abad-Fuentes, A. Novel haptens and monoclonal antibodies with subnanomolar affinity for a classical analytical target, ochratoxin A. *Sci Rep* **2018**, *8*, 9761.

(2) López-Puertollano, D.; Agulló, C.; Mercader, J. V.; Abad-Somovilla, A.; Abad-Fuentes, A. Immunoanalytical methods for ochratoxin A monitoring in wine and must based on innovative immunoreagents. *Food Chem* **2021**, *345*, 128828.

(3) Hülagü, D.; Tobias, C.; Climent, E.; Gojani, A.; Rurack, K.; Hodoroaba, V. D. Generalized Analysis Approach of the Profile Roughness by Electron Microscopy with the Example of Hierarchically Grown Polystyrene-Iron Oxide-Silica Core-Shell-Shell Particles. *Adv Eng Mater* **2022**, *24*, 2101344.

(4) Tobias, C.; Lopez-Puertollano, D.; Abad-Somovilla, A.; Mercader, J.; Abad-Fuentes, A.; Rurack, K. Development of Simple and Rapid Bead-Based Cytometric Immunoassays Using Superparamagnetic Hybrid Core-Shell Microparticles. *ACS Meas Sci Au* **2024**, *4*, 678–688.

(5) Hülagü, D.; Tobias, C.; Dao, R.; Komarov, P.; Rurack, K.; Hodoroaba, V.-D. Towards 3D determination of the surface roughness of core–shell microparticles as a routine quality control procedure by scanning electron microscopy. *Sci Rep* **2024**, *14*, 17936.

(6) López-Puertollano, D.; Duncan, H.; Abad-Somovilla, A.; Abad-Fuentes, A.; Rurack, K. Competitive cytometry-based immunoassay for patulin determination in apple juice. *Microchem J* **2025**, *212*, 113287.

(7) Tobias, C.; Climent, E.; Gawlitza, K.; Rurack, K. Polystyrene Microparticles with Convergently Grown Mesoporous Silica Shells as a Promising Tool for Multiplexed Bioanalytical Assays. *ACS Appl Mater Inter* **2021**, *13*, 207-218.

(8) Li, J. J.; Ouellette, A. L.; Giovangrandi, L.; Cooper, D. E.; Ricco, A. J.; Kovacs, G. T. A. Optical Scanner for Immunoassays With Up-Converting Phosphorescent Labels. *IEEE Trans Biomed Eng* **2008**, *55*, 1560-1571.

(9) Parsa, M. K.; Hormozi, F. Experimental and CFD modeling of fluid mixing in sinusoidal microchannels with different phase shift between side walls. *J Micromech Microeng* **2014**, *24*, 065018.

(10) Rendl, M.; Brandstetter, T.; Rühe, J. Time-Resolved Analysis of Biological Reactions Based on Heterogeneous Assays in Liquid Plugs of Nanoliter Volume. *Anal Chem* **2013**, *85*, 9469-9477.

(11) Gupta, A.; Yan, D. S. Magnetic and Electrostatic Separation. In *Mineral Processing Design and Operations*, 2nd ed.; Elsevier, 2016; pp 629-687.

(12) Abdelshafi, N. A.; Bell, J.; Rurack, K.; Schneider, R. J. Microfluidic electrochemical immunosensor for the trace analysis of cocaine in water and body fluids. *Drug Test Anal* **2019**, *11*, 492-500.

(13) Alam, M. *DIY SmartPhone Oscilloscope using Raspberry Pi Pico*. 2023. https://how2electronics.com/diy-smartphone-oscilloscope-using-raspberry-pi-pico/ (accessed 04/2025).

(14) Annunziata, L.; Schirone, M.; Visciano, P.; Campana, G.; De Massis, M. R.; Migliorati, G. Determination of aflatoxins, deoxynivalenol, ochratoxin A and zearalenone in organic wheat flour under different storage conditions. *Int J Food Sci Technol* **2021**, *56*, 4139-4148.

(15) Elaridi, J.; Yamani, O.; Al Matari, A.; Dakroub, S.; Attieh, Z. Determination of Ochratoxin A (OTA), Ochratoxin B (OTB), T-2, and HT-2 Toxins in Wheat Grains, Wheat Flour, and Bread in Lebanon by LC-MS/MS. *Toxins* **2019**, *11*, 471.

(16) Sun, C. N.; Liao, X. F.; Huang, P. X.; Shan, G. Z.; Ma, X.; Fu, L. Z.; Zhou, L. D.; Kong, W. J. A self-assembled electrochemical immunosensor for ultra-sensitive detection of ochratoxin A in medicinal and edible malt. *Food Chem* **2020**, *315*, 126289.

(17) Zhou, J.; Yang, Q.; Liang, C.; Chen, Y.; Zhang, X.; Liu, Z.; Wang, A. Detection of ochratoxin A by quantum dots–based fluorescent immunochromatographic assay. *Anal Bioanal Chem* **2021**, *413*, 183-192.

(18) Zhang, M.; Zhang, S.; Guo, X.; Xun, Z.; Wang, L.; Liu, Y.; Mou, W.; Qin, T.; Xu, Z.; Wang, L.; et al. Fast, portable, selective, and ratiometric determination of ochratoxin A (OTA) by a fluorescent supramolecular sensor. *J Hazard Mater* **2024**, *465*, 133104.

(19) Machado, J. M. D.; Soares, R. R. G.; Chu, V.; Conde, J. P. Multiplexed capillary microfluidic immunoassay with smartphone data acquisition for parallel mycotoxin detection. *Biosens Bioelectron* **2018**, *99*, 40-46.

(20) Hao, N.; Dai, Z.; Meng, X.; Hua, R.; Lu, J.; Wang, K. A portable solar-driven ratiometric photo-electrochromic visualization biosensor for detection of ochratoxin A. *Sens Actuators, B* **2020**, *306*, 127594.

(21) Feng, X.; Yuan, R.; Liu, L.; Ding, L.; Long, L.; Wang, K. Construction of dual-signal output sensing platform for different scene of rapid and sensitive ochratoxin A detection in corn. *Talanta* **2025**, *282*, 126991.

(22) Sang, M.; Meng, X.; Zhang, Y.; Li, Z.; Zhou, Q.; Jing, X.; Sun, X.; Zhao, W. An “on–off–on” electrochemiluminescence aptasensor based on a self-enhanced luminophore for ochratoxin A detection. *Anal Bioanal Chem* **2023**, *415*, 5833-5844.

(23) Karachaliou, C. E.; Koukouvinos, G.; Zisis, G.; Kizis, D.; Krystalli, E.; Siragakis, G.; Goustouridis, D.; Kakabakos, S.; Petrou, P.; Livaniou, E.; et al. Fast and Accurate Determination of Minute Ochratoxin A Levels in Cereal Flours and Wine with the Label-Free White Light Reflectance Spectroscopy Biosensing Platform. *Biosensors-Basel* **2022**, *12*, 877.

(24) Chen, Y.; Meng, X.; Zhu, Y.; Shen, M.; Lu, Y.; Cheng, J.; Xu, Y. Rapid detection of four mycotoxins in corn using a microfluidics and microarray-based immunoassay system. *Talanta* **2018**, *186*, 299-305.

(25) Soares, R. R. G.; Santos, D. R.; Pinto, I. F.; Azevedo, A. M.; Aires-Barros, M. R.; Chu, V.; Conde, J. P. Multiplexed microfluidic fluorescence immunoassay with photodiode array signal acquisition for sub-minute and point-of-need detection of mycotoxins. *Lab Chip* **2018**, *18*, 1569-1580.

(26) Costantini, F.; Lovecchio, N.; Ruggi, A.; Manetti, C.; Nascetti, A.; Reverberi, M.; de Cesare, G.; Caputo, D. Fluorescent Label-Free Aptasensor Integrated in a Lab-on-Chip System for the Detection of Ochratoxin A in Beer and Wheat. *ACS Appl Bio Mater* **2019**, *2*, 5880-5887.

(27) Höfs, S.; Hülagü, D.; Bennet, F.; Carl, P.; Flemig, S.; Schmid, T.; Schenk, J. A.; Hodoroaba, V. D.; Schneider, R. J. Electrochemical Immunomagnetic Ochratoxin A Sensing: Steps Forward in the Application of 3,3',5,5'-Tetramethylbenzidine in Amperometric Assays. *ChemElectroChem* **2021**, *8*, 2597-2606.

(28) Zangheri, M.; Di Nardo, F.; Calabria, D.; Marchegiani, E.; Anfossi, L.; Guardigli, M.; Mirasoli, M.; Baggiani, C.; Roda, A. Smartphone biosensor for point-of-need chemiluminescence detection of ochratoxin A in wine and coffee. *Anal Chim Acta* **2021**, *1163*, 338515.

(29) *Evaluation of Measurement Data—Guide to the Expression of Uncertainty in Measurement*; Joint Committee for Guides in Metrology JCGM, Paris, 1 Edn, 2008, corrected version 2010.

(30) Bich, W.; Cox, M. G.; Harris, P. M. Evolution of the 'Guide to the Expression of Uncertainty in Measurement'. *Metrologia* **2006**, *43*, S161.
